# Natural History and Treatment Outcomes of Congenital Thrombotic Thrombocytopenic Purpura: A Retrospective Longitudinal Cohort Study

**DOI:** 10.36469/001c.164543

**Published:** 2026-08-12

**Authors:** Paul Coppo, Marie Scully, Johanna A. Kremer Hovinga, Bérangère S. Joly, Agnès Veyradier, Ping Du, Parth Patwari, Linda T. Wang, Björn Mellgård, Hamid Ferdosi, Ragy Saad

**Affiliations:** 1 Department of Hematology and National Reference Center for Thrombotic Microangiopathies Hôpital Saint-Antoine, Assistance Publique-Hôpitaux de Paris and Sorbonne – Université (AP-HP.6), Paris, France; 2 Université Paris Cité, Sorbonne Université, Inserm UMRS_1138, Centre de Recherche des Cordeliers, Paris, France; 3 Department of Haematology University College London Hospital, Haematology Theme-NIHR UCLH/UCL BRC, London, UK; 4 Department of Hematology and Central Hematology Laboratory Bern University Hospital, University of Bern, Bern, Switzerland; 5 Department of Biological Hematology AP-HP.Nord, Lariboisière hospital, Paris, France; 6 Takeda Development Center Americas, Inc., Cambridge, Massachusetts, USA; 7 Department of Epidemiology, Milken Institute School of Public Health George Washington University, Washington, DC, USA

**Keywords:** ADAMTS13, cTTP, congenital thrombotic thrombocytopenic purpura, prophylaxis, real-world evidence

## Abstract

**Background:**

Congenital thrombotic thrombocytopenic purpura (cTTP) is an ultra-rare, life-threatening thrombotic disorder. The real-world clinical trajectory and management of cTTP are not well defined.

**Objectives:**

Describe patient characteristics, quantify the incidence and prevalence of clinical manifestations and disease-related complications, and describe treatment patterns and treatment-related outcomes of patients with cTTP.

**Methods:**

In this retrospective, multinational, longitudinal cohort study, data were abstracted from the medical records of patients with severe hereditary ADAMTS13 deficiency (<10% activity) at 9 participating sites across Europe and the United States. Eligible patients experienced an index event (cTTP diagnosis, an acute TTP event or TTP manifestation, a cTTP-related clinical event, or prophylaxis) between January 1, 2009, and December 31, 2017. Data collection was from January 1, 2009, to December 31, 2020. A post hoc analysis was conducted among patients who matched inclusion criteria for a phase 3 trial (NCT03393975), and who received plasma-based therapy (PBT) prophylaxis at least once per month, up to 3 times per week.

**Results:**

Medical records from 78 patients (61 [78.2%] female) were included in the study. The mean (SD) follow-up was 8.1 (3.1) years. Ninety-two acute TTP events were recorded in 55 (70.5%) patients (overall event rate, 0.145 events per person-year). Eighty (87.0%) acute TTP events occurred in the absence of prophylactic treatment, of which 16 (20.0%) resulted in organ damage (based on physicians’ assessment). Twelve (13.0%) acute TTP events occurred during prophylaxis; none resulted in organ damage. Sixty-four TTP manifestations were recorded in 29 (37.2%) patients (overall event rate, 0.101 events per person-year). Of the 25 patients in the post hoc analysis, 4 experienced 9 acute TTP events while receiving PBT prophylaxis.

**Conclusions:**

Our findings suggest that acute TTP events occurred less frequently during periods of prophylaxis but also highlight prophylaxis’ intrinsic limitations in preventing ongoing manifestations, cumulative organ damage, and the logistical and safety burdens associated with repeated plasma infusions. Patients with cTTP bear a substantial clinical and disease burden associated with acute TTP events and organ damage. Despite treatment with PBT prophylaxis, some patients still experienced acute TTP events, suggesting that more effective treatments may be needed.

## INTRODUCTION

Congenital thrombotic thrombocytopenic purpura (cTTP) is an ultra-rare, life-threatening, and chronic thrombotic disorder of the microcirculation caused by inherited deficiency of a disintegrin and metalloproteinase with thrombospondin motifs 13 (ADAMTS13).^1–3^ The diagnosed prevalence of cTTP has been estimated at 0.5 to 2 cases per million.^4^ However, based on a 2024 population-based genetic epidemiology study, cTTP prevalence may be higher than previously understood.^5^ Although cTTP can present at any age, 2 peak periods of presentation have been observed: early childhood and adulthood, particularly in the context of pregnancy.^6^

Clinically, cTTP manifests as acute thrombotic thrombocytopenic purpura (TTP) events characterized by thrombocytopenia, microangiopathic hemolytic anemia, and ischemic organ damage that can include neurological, cardiac, gastrointestinal, and renal involvement.^6–8^ Pregnancy and infections are typical precipitating factors for acute TTP events.^7,9^ In addition, patients can experience debilitating chronic or intermittent non-overt symptoms such as headache, migraine (with or without auras), abdominal pain, and fatigue. These symptoms may reflect ongoing disease activity due to persistent ADAMTS13 deficiency, which may be associated with cumulative organ injury over time.^6–8^ Treatment for cTTP has focused on ADAMTS13 replacement via plasma-based therapies (PBTs). Two preparations are commonly used: fresh frozen plasma (FFP) and solvent/detergent-treated plasma.^6,10–14^

Despite a growing consensus among international working groups on the diagnosis of cTTP and the management of acute TTP events,^13,15^ substantial evidence gaps remain in our understanding of the natural history (including periods with and without prophylactic treatment), clinical burden, and long-term sequelae. Existing data are largely derived from single-country registries, or clinical trial populations, and often focus on acute presentations rather than the cumulative burden of disease and treatment over time. Real-world evidence from a broader multinational cohort may facilitate the development of accurate disease models and inform treatment strategy, including the integration of novel targeted therapies, with the aim of reducing and mitigating the clinical burden of cTTP and improving patient outcomes.

The aim of this study was to address key knowledge gaps in the health impacts and real-world management of patients living with cTTP. The primary objectives were to describe the characteristics of patients with cTTP, and to quantify the incidence and prevalence of short- and long-term clinical manifestations (acute TTP events and TTP manifestations), and disease-related complications. Secondary objectives included describing the treatment patterns and treatment-related outcomes of patients with cTTP. Findings from this study expand prior knowledge with data from a multinational cohort with extended longitudinal follow-up, and by including TTP manifestations and real-world impacts beyond acute TTP events. Additionally, endpoints were selected to align with a prior clinical trial,^16^ allowing for better translation of clinical trial results into real-world global settings. This information may help to inform therapeutic decision-making and identify priorities for future research.

## METHODS

### Study Design

This was a multinational, retrospective, longitudinal cohort study using de-identified medical records from 9 participating sites: France (1 site), the United Kingdom (2), Spain (2), Switzerland (1), Germany (1), Italy (1), and the United States (1). Institutions were invited to participate in the study based on convenience sampling from the target population (ie, patients with a confirmed diagnosis of cTTP). Convenience sampling was considered necessary given the ultra-rarity of cTTP and to enable a meaningful sample size to be reached (see **Methods: Statistical Analysis Plan**). Site selection considered physicians’ expertise in managing cTTP, eligible patient volume, prior research experience, and capacity to conduct chart reviews. Participating sites were mainly specialty treatment centers. Trained personnel at each participating site abstracted patient-level data under the supervision of clinicians specializing in thrombotic microangiopathies including cTTP, using a web-based electronic case report form on an electronic data capture platform. Collected variables and timepoints were standardized across sites using an electronic data capture form (**Supplementary Materials: Statistical Analysis Plan)**. Centralized remote monitoring was implemented on all data entered into the electronic case report form. On-site monitoring visits were also conducted.

Data were collected from medical records during the study period (January 1, 2009, to December 31, 2020) for eligible patients who had at least 1 qualifying index event (detailed in **Supplementary Table S1**) during the index identification period (January 1, 2009, to December 31, 2017). Qualifying index events included: an initial diagnosis of cTTP, an acute TTP event, TTP manifestation, a TTP-related clinical event, or receipt of prophylaxis, as defined in **Supplementary Table S1**. The index date was defined as the date of the earliest qualifying event. Patients were followed from their study index date until loss to follow-up, enrollment in a clinical trial, death, or the end of the study period, whichever occurred first (**Supplementary Figure S1**).

### Patient Population

The study was conducted in accordance with the Declaration of Helsinki and followed the Guidelines for Good Pharmacoepidemiology Practices, recommended by the International Society for Pharmacoepidemiology. Institutional review board/independent ethics committee approval was obtained from each participating study site as required by local regulations. Written informed consent or a waiver of informed consent was obtained as applicable and in accordance with the requirements of the relevant ethical committees in each country.

Eligible patients had a documented diagnosis of severe hereditary ADAMTS13 deficiency (defined in **Supplementary Table S1**). Exclusion criteria included a prior diagnosis of any other hematologic disorder, except for hemolytic uremic syndrome. Results were stratified based on age at diagnosis and further stratified by cTTP diagnosis occurring during or outside of pregnancy among adult-diagnosed patients. A post hoc analysis was conducted to generate real-world evidence on the burden of cTTP to support the assessment of efficacy and safety of the use of rADAMTS13 for patients with cTTP by health authorities. This analysis included the subset of all patients who would have met eligibility criteria for its phase 3 pivotal trial (NCT03393975; **Supplementary Methods**).^16^ Briefly, patients in the post hoc analysis were 0-70 years old, and received regular prophylaxis (defined as treatment administered at a frequency of ≥1 per month and ≤3 per week, excluding steroids) with PBT. Exclusion criteria included pregnancy (for females ≥14 years old), drug dependence, dementia, Alzheimer’s disease, alcohol dependence, HIV, cirrhosis, or acute coronary syndrome.

### Study Variables

Baseline characteristics recorded based on information available on or before study index included sociodemographic variables, general health factors, and cTTP-related characteristics, including diagnostic history with ADAMTS13 phenotypic and genotypic investigations (**Supplementary Methods**). Clinical manifestations of TTP were classified as acute TTP events or TTP manifestations. An acute TTP event was defined as a decrease in platelet count of at least 50% of baseline or a platelet count of fewer than 100 000/mL and an elevation of lactate dehydrogenase more than twice the upper limit of normal. A TTP manifestation was defined as an event that met any of the following: a decrease in platelet count of at least 25% of baseline at screening or a platelet count of fewer than 150 000/mL; elevation of lactate dehydrogenase more than 1.5 times the upper limit of normal; and/or relevant signs and symptoms as defined in **Supplementary Table S1**. Situations that precipitated acute TTP events during the study period were recorded.

Disease-related complications included organ damage and dysfunction, symptomatology of cTTP, and death. Organ damage was described overall and classified by organ type: cardiac, neurological, renal, or hepatic. Organ dysfunction recorded during annual organ assessments was described overall and by organ system (cardiac, renal, neurological, and hepatic). Treatment patterns and treatment-related outcomes for acute TTP events during the study period and TTP manifestations at study index were also recorded. Other variables were collected as per **Supplementary Materials: Statistical Analysis Plan**.

### Statistical Analysis

All analyses were descriptive, and no formal hypothesis testing or inferential statistical analyses were performed. No imputations of missing data were conducted. Categorical variables are presented as frequencies and percentages. Continuous variables are expressed as the mean, median, SD, IQR, and range. For instances in which patients had repeat observations, within-patient averages were calculated.

Prevalence was calculated as the number of patients who ever experienced an event divided by the total number of eligible patients. Prevalence was expressed as N (%) or per 100 persons for acute TTP events, TTP manifestations, organ damage (at acute TTP events during the study period and at TTP manifestations at index), and organ dysfunction. Incidence rates were expressed as rates of event(s) per person-year (PPY) and assessed among patients who had no history of the event of interest before study index. Patients with sufficient baseline history to exclude prevalent cases comprised the at-risk population for deriving incidence rates. Patients contributed follow-up time from their index date until study end or censoring, either due to the reasons above or the occurrence of the event of interest. Event rates (ERs) were calculated for acute TTP events and TTP manifestations during the study period. ERs for acute TTP events occurring during prophylaxis considered only the time period in which patients received prophylaxis and the events occurring in that period. Similarly, ERs for acute TTP events occurring outside of prophylaxis considered only time periods without prophylaxis and the events occurring in that period. Patients could have had more than one ER if they had periods with and without prophylaxis.

**Equation 1:**
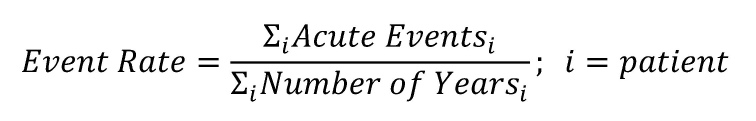


In the post hoc analysis, ERs were calculated from the subset of events that occurred while patients were receiving PBT prophylaxis among all person-years on regular prophylaxis.

**Equation 2:**
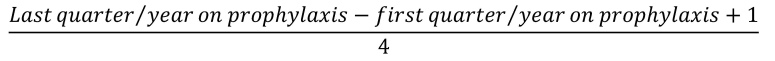


Given the descriptive nature of this study, no formal sample-size calculations were performed. To strengthen the interpretability of the results, we targeted 80 patients for inclusion using convenience sampling of all consecutive eligible patients across participating sites (country targets in **Supplementary Methods** and **Supplementary Table S2**). All analyses were conducted using baseline packages for statistical analysis in STATA version 17 (StataCorp LLC). Incomplete or missing information was documented for all study variables where applicable. Missing data were not imputed. The reporting of this study conforms to the Strengthening the Reporting of Observational Studies in Epidemiology (STROBE) statement.^17^

## RESULTS

### Primary Outcomes

**Patient demographics and clinical characteristics**: Forty-two sites across 10 countries were invited to participate in this study. Seventy-eight patients from 9 sites in 7 countries were included in this study. Of these 78 patients, 61 (78.2%) were female and 50 (64.1%) were from France (**Table 1**). Due to the high proportion of participants from France, some results highlight the France-specific analysis alongside the overall data. The median (range) age at study index was 28.0 (1-82) years. The mean (SD) duration of follow-up was 8.1 (3.1) years. The median (IQR) age at diagnosis was 26.5 (14.5-37.0) years (**Table 1**). Diagnosis occurred during childhood (<18 years of age) in 21 (26.9%) patients. Of the 57 (73.1%) patients who were diagnosed as adults (≥18 years of age), 30 (52.6%) were diagnosed during or immediately following pregnancy. Patients from France had generally similar baseline characteristics to the overall study population, except that patients tended to be diagnosed at an earlier age, with a smaller proportion of patients diagnosed at ages ≥45 years (**Supplementary Table S3**).

**Table 1.** Demographics, Clinical Characteristics, and Index Events for All Patients and for Those Included in the Post Hoc Analysis^a^

**Characteristics**	**All Patients (N = 78)**	**Post Hoc Analysis (n = 25)**
Sex, n (%)		
Female	61 (78.2)	17 (68)
Male	17 (21.8)	8 (32)
Median (IQR) [range] age at study index, y	28.0 (21-37.75) [1-82]	23.0 (13-36) [1-67]
Age range at index event, n (%)		
<18 y	14 (18.0)	10 (40)
18-29 y	28 (35.9)	6 (24)
30-44 y	25 (32.1)	5 (20)
45-64 y	8 (10.3)	3 (12)
≥65 y	3 (3.9)	1 (4)
Median (IQR) [range] age at diagnosis, y	26.5 (14.5-37.0) [0-67]	8.0 (2.8-33.0) [0-65.3]
Age range at diagnosis, n (%)		
<18 y	21 (26.9)	16 (64)
18-29 y	25 (32.1)	2 (8)
30-44 y	21 (26.9)	3 (12)
45-64 y	9 (11.5)	3 (12)
≥65 y	2 (2.6)	1 (4)
≥18 y during pregnancy	30 (38.5)	-
≥18 y outside of pregnancy	27 (34.6)	-
Type of index event, n (%)^b^		
Prophylactic treatment	16 (20.5)	8 (32)
cTTP diagnosis	35 (44.9)	8 (32)
Acute TTP event	18 (23.1)	4 (16)
TTP manifestation	4 (5.1)	4 (16)
cTTP-related clinical event	5 (6.4)	1 (4)
Country, n (%)		
France	50 (64.1)	17 (68.0)
Germany	5 (6.4)	0
Italy	2 (2.6)	1 (4.0)
Spain	6 (7.7)	0
Switzerland	6 (7.7)	2 (8.0)
United Kingdom	8 (10.3)	4 (16.0)
United States	1 (1.3)	1 (4.0)
Race/ethnicity, n (%)		
White/Caucasian	25 (32.1)	7 (28)
Black/Afro-Caribbean	2 (2.6)	1 (4)
Hispanic/Latino	1 (1.3)	0
Missing	50 (64.1)	17 (68)
General health risk factors, n (%)^c^		
Underweight (BMI <18.5)	6 (9.1)	-
Overweight (BMI 25 to <30)	15 (22.7)	-
Obese (BMI ≥30)	8 (12.1)	-
Current smoker	12 (15.4)	-
Former smoker	6 (7.7)	-
Drug dependence	2 (3.3)	-
Alcohol dependence	0	-

Abbreviations: BMI, body mass index (kg/m^2^); cTTP, congenital thrombotic thrombocytopenic purpura; TTP, thrombotic thrombocytopenic purpura.

Information to calculate BMI was missing for 12 patients.

^a^The post hoc analysis focused on the subset of patients who had similar characteristics to those who received plasma-based therapy prophylaxis in a phase 3 pivotal trial (NCT03393975).

^b^Index event definitions are provided in **Supplementary Table S1**.

^c^Denominators are 66 for BMI (adult patients aged ≥20 years), 78 for smoking status, and 60 for drug and alcohol dependence (patients with available data).

**Figure 1.**
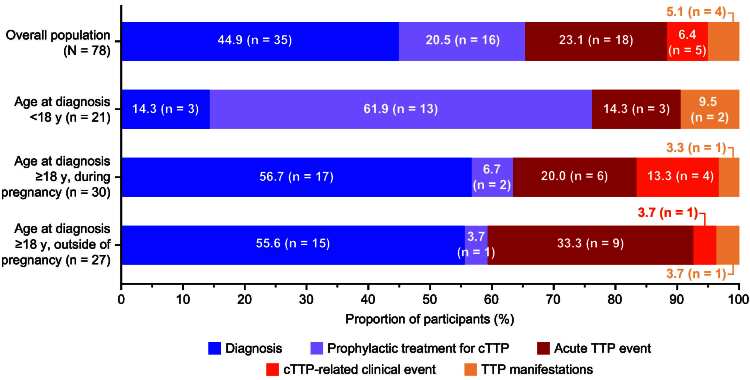
Index Events by Age Abbreviations: cTTP, congenital thrombotic thrombocytopenic purpura; TTP, thrombotic thrombocytopenic purpura. Among the 35 patients for whom cTTP diagnosis was the index event, 28 (80.0%) had a concurrent acute TTP event. Diagnosis, acute TTP events, cTTP-related clinical events, and isolated TTP manifestations were defined as per **Supplementary Table S1**.

Initial cTTP diagnosis was the most common index event in the overall study population (**Table 1**), and among patients from France and Switzerland (**Supplementary Table S4**). cTTP diagnosis, or cTTP diagnosis corresponding with an acute TTP event, were the most common index events in Spain and the UK. An acute TTP event was the most common index event in Germany. In the overall population, prophylaxis initiation was the most common index event among patients who were diagnosed in childhood (13/21, 61.9%; **Figure 1**), whereas for those who were diagnosed in adulthood, the initial cTTP diagnosis was the most common index event (32/57, 56.1%). In the overall study population, 47/78 (60.3%) patients received any prophylaxis (**Supplementary Table S5**). Most patients from France, the UK, and the US received prophylaxis (34/50 [68.0%], 7/8 [87.5%], and 1/1 [100%], respectively), whereas none of the 5 patients from Germany received prophylaxis.

**Figure 2.**
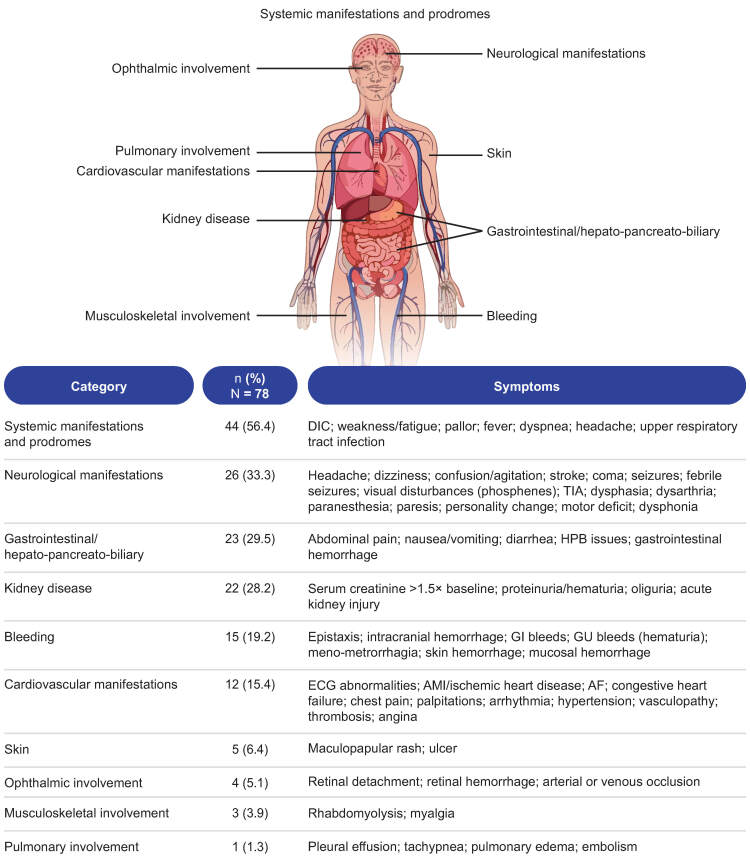
Clinical Signs and Symptoms at cTTP Diagnosis Abbreviations: AF, atrial fibrillation; AMI, acute myocardial infarction; cTTP, congenital thrombotic thrombocytopenia; DIC, disseminated intravascular coagulation; ECG, electrocardiogram; GI, gastrointestinal; GU, genitourinary; HPB, hepato-pancreato-biliary; TIA, transient ischemic attack. Patients could experience more than 1 symptom.

The diagnosis of cTTP was confirmed for all patients; for most patients, confirmation was based on severe ADAMTS13 deficiency (activity <10 IU/dL, n = 67, 85.9%) and/or genetic sequencing (n = 49, 62.8%). Six (7.7%) patients had a known family history of cTTP. Systemic manifestations and prodromes were common at initial presentation (n = 44, 56.4%; **Figure 2**). Other common clinical presentations at diagnosis were neurological manifestations (n = 26, 33.3%), gastrointestinal/hepato-pancreato-biliary manifestations (n = 23, 29.5%), and kidney disease (n = 22, 28.2%).

Comorbidity information was available for 60 of 78 patients. Among these 60 patients with comorbidity data, 43 (71.7%) had at least 1 co-occurring comorbidity; depression (8/60, 13.3%) and anxiety (4/60, 6.7%) were the most frequent. Cardiovascular disorders and risk factors such as hypertension and hypercholesterolemia were recorded in 5 (8.3%) and 3 (5.0%) patients, respectively. Two (3.3%) patients had a history of stroke (information on type not provided).

**cTTP clinical manifestations: Acute TTP events**: During follow-up, 92 acute TTP events were recorded in 55 (70.5%) patients. The majority of events (80 events, 87.0%) occurred in the absence of prophylactic treatment, whereas 12 (13.0%) events occurred during prophylaxis exposure periods (**Table 2**). The mean (SD) number of acute TTP events per patient was 1.7 (1.5) and the overall ER for acute TTP events was 0.145 events PPY. ERs were similar for patients who were younger than 18 years old and those 18 years or older at diagnosis (0.125 and 0.155 events PPY, respectively). The rate of acute TTP events among adult patients who were diagnosed outside pregnancy was 0.232 events PPY, compared with 0.094 events PPY for patients diagnosed during or immediately after pregnancy. The ER was 0.050 events PPY (n = 47) during periods of prophylaxis and 0.202 events PPY (n = 70) during periods without prophylaxis. For country-specific context, the ER for acute TTP events for patients from France was 0.095 events PPY, compared with 0.145 events PPY for the overall study population.

**Table 2.** Event Rates of cTTP Clinical Manifestations

	**Patients^a^, n**	**Person-Years^b^**	**cTTP Clinical Manifestation**		
**Patients Who Had ≥1 Event, n (%)**	**Events, n**	**Event Rate, PPY**			
**Acute TTP Events—Total**	78	634.5	55 (70.5)	92	0.145
cTTP prophylaxis during study period^c^					
Prophylaxis^d^	47	238.5	6 (12.8)	12	0.050
No prophylaxis	70	396.0	50 (71.4)	80	0.202
Sex					
Female	61	497.0	44 (72.1)	63	0.127
Male	17	137.5	11 (64.7)	29	0.211
Age at index, y					
<18	14	149.3	6 (42.9)	21	0.141
18-29	28	233.5	20 (71.4)	23	0.099
30-44	25	184.8	19 (76.0)	28	0.152
45-64	8	54.3	7 (87.5)	17	0.313
≥65	3	12.8	3 (100)	3	0.235
Age at diagnosis, y					
<18	21	216.3	9 (42.9)	27	0.125
≥18 during pregnancy	30	233.0	21 (70.0)	22	0.094
≥18 outside pregnancy	27	185.3	25 (92.6)	43	0.232
Time since diagnosis at index, y					
≤1^c^	56	424.3	46 (82.1)	72	0.170
>1 to ≤5	7	79.0	1 (14.3)	4	0.051
>5 to ≤10	7	78.3	3 (42.9)	7	0.089
>10	8	53.0	5 (62.5)	9	0.170
Diagnosis year					
2008 or earlier	23	221.8	9 (39.1)	20	0.090
2009 or later	55	412.8	46 (83.6)	72	0.174
Prior history of acute TTP events^d^					
No acute TTP events before study	58	442.5	46 (79.3)	72	0.163
Acute TTP events before study	20	192.0	9 (45.0)	20	0.104
**TTP Manifestations—Total**	78	634.5	29 (37.2)	64	0.101
Sex					
Female	61	497.0	21 (34.4)	45	0.091
Male	17	137.5	8 (47.1)	19	0.138
Age at index, y					
<18	14	149.3	8 (57.1)	21	0.141
18-29	28	233.5	10 (35.7)	22	0.094
30-44	25	184.8	7 (28.0)	9	0.049
45-64	8	54.3	4 (50.0)	12	0.221
≥65	3	12.8	0	0	0.000
Time since diagnosis at index^e^, y					
≤1	56	424.3	19 (33.9)	38	0.090
>1 to ≤5	7	79.0	4 (57.1)	6	0.076
>5 to ≤10	7	78.3	3 (42.9)	8	0.102
>10	8	53.0	3 (37.5)	12	0.226
Diagnosis year					
2008 or earlier	23	221.8	11 (47.8)	27	0.122
2009 or later	55	412.8	18 (32.7)	37	0.090
Prior history of acute TTP events^f^					
No acute TTP events before study	58	442.5	21 (36.2)	40	0.090
Acute TTP events before study	20	192.0	8 (40.0)	24	0.125

Abbreviations: cTTP, congenital thrombotic thrombocytopenic purpura; PPY, per person-year; TTP, thrombotic thrombocytopenic purpura.

^a^For number and type of treatment received, n refers to the number of patients who had 1 or fewer events.

^b^For each patient, follow-up corresponds to the time between the index and end of study or death, both inclusive. For example, a patient with index in quarter 2 of 2015 who did not die before the end of the study (quarter 4 of 2020) contributed 5.75 person-years in the study.

^c^Numbers of patients in subgroups do not sum to total (N = 78), as a single patient could have events both while receiving prophylaxis and while not receiving prophylaxis.

^d^For prophylaxis, only the period in which patients had any prophylaxis (and the events that occurred in that period) was counted. If patients also had a period without prophylaxis, data were counted in the “no prophylaxis” row.

^e^Includes patients whose index date preceded their diagnosis.

^f^History of acute TTP events prior to the study period was only available for 23 patients diagnosed before the study period, among whom the majority (n = 20) had acute TTP events recorded before the study period.

Most acute TTP events (70 events, 76.1%) resolved without complications. However, 4 (4.3%) resolved with short-term complications (based on the discretion of the treating physician), 16 (17.4%) resolved but resulted in organ damage (see below), and 2 (2.2%) resulted in death. Platelet normalization (platelet count >150 000/µL) occurred for 79 (85.9%) acute TTP events, with counts normalizing within 7 days for 26 (32.9%) events, within 8 to 14 days for 14 (17.7%) events, and after more than 14 days for 12 (15.2%) events. Platelet count normalization data were missing for 27 acute TTP events.

Precipitating factors were recorded for 46 acute TTP events, of which infection (n = 21, 45.7%) and pregnancy (n = 17, 37.0%) were the most common. Presenting symptoms at the time of an acute TTP event were recorded for 55 patients and were similar to those recorded at cTTP diagnosis, with neurological manifestations (n = 17, 30.9%), systemic manifestations and prodromes (n = 16, 29.1%), and gastrointestinal/hepato-pancreato-biliary manifestations (n = 13, 23.6%) most frequently recorded.

**cTTP clinical manifestations: TTP manifestations:** Overall, 64 TTP manifestations were recorded in 29 (37.2%) patients, yielding a rate of 0.101 events PPY (**Table 2**)—lower than the 92 acute TTP events in 55 patients (0.145 events PPY). The incidence rate of TTP manifestations was not known, as information on events occurring before study entry was not available for all patients. The majority (43/64, 67.2%) of the TTP manifestations were recorded in patients aged younger than 30 years at study index. The mean (SD) number of TTP manifestations per patient was 2.2 (2.4). For country-specific context, the ER for TTP manifestations for patients from France was 0.090 events PPY, compared with 0.101 events PPY for the overall study population.

**Figure 3.**
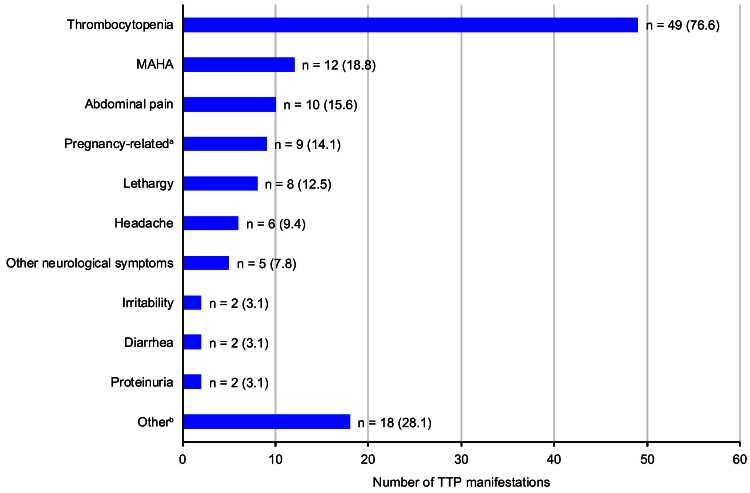
Clinical Presentation of TTP Manifestations (N = 64) Abbreviations: TTP, thrombotic thrombocytopenic purpura; MAHA, microangiopathic hemolytic anemia. Patients could experience more than 1 sign/symptom during a TTP manifestation. ^a^Other: bruise (n = 1); fatigue, general malaise (n = 1); febricula, asthenia, intrabuccal hemorrhagic bubbles (n = 1); fever (n = 1); hematomas (n = 1); petechiae (n = 5); petechiae bruising (n = 1); right posterior sylvian infarction (n = 1); swelling and tingling of extremities (n = 1); weariness (n = 1); missing information (n = 1); motor symptoms (n = 1); increased creatinine (n = 1); dysarthria (n = 1).

Thrombocytopenia (defined in **Supplementary Table S1**) was the most common clinical presentation for TTP manifestations (n = 49, 76.6%; **Figure 3**), followed by microangiopathic hemolytic anemia (n = 12, 18.8%; defined in **Supplementary Table S1**), and abdominal pain (n = 10, 15.6%). Pregnancy was associated with 9 (14.1%) TTP manifestations. Nearly all (n = 58, 90.6%) TTP manifestations resolved clinically, with 16 (27.6%) resolving within 7 days, 7 (12.1%) resolving within 8 to 14 days, and 1 (1.7%) resolving after more than 14 days. However, time-to-resolution data were missing for 34 TTP manifestations.

**cTTP disease-related complications:** Organ damage was reported in 22 (28.2%) patients who experienced 32 acute TTP events during follow-up. The overall ER for organ damage temporally associated with acute TTP events occurring during follow-up was 0.050 events PPY. Most acute TTP events associated with organ damage occurred in patients aged 30 to 64 years at study index (19/32 events, 59.4%). Patients whose study index date occurred up to and including 1 year since their initial cTTP diagnosis had the highest ER for organ damage (0.064 events PPY). Fifteen (19.2%) patients experienced neurological organ damage at the time of their acute TTP event; 11 (14.1%), 8 (10.3%), and 3 (3.8%) experienced kidney disease, cardiac damage, and liver damage, respectively. Among the 80 acute TTP events that occurred in the absence of prophylaxis, 16 (20.0%) resulted in organ damage. None of the 12 acute TTP events that occurred during prophylaxis led to organ damage. For country-specific context, patients from France, the overall ER for organ damage recorded during acute TTP events was 0.030 events PPY, compared with 0.050 events PPY for the overall study population.

Of the 15 patients who experienced more than 1 acute TTP event, 5 experienced organ damage: cardiac damage (3), neurological damage (4), kidney damage (2), and liver damage (1). Additionally, 22 patients underwent annual organ assessments during the study period. Six (27.3%) patients reported an organ dysfunction. Ischemia was the most commonly recorded type of organ dysfunction, affecting 5 (22.7%) patients. No liver impairment was recorded.

### Secondary Outcomes

**Treatment patterns in cTTP**: In total, 52 patients received treatment for 1 or more acute TTP events, with 32 (61.5%) patients receiving 1 treatment for 1 or more of these events (**Table 3**). The most commonly administered treatments were plasma exchange (n = 30, 57.7%) and FFP (n = 25, 48.1%). Treatments administered for the 4 TTP manifestations that occurred at study index included plasma exchange, plasma treated with a solvent/detergent process, and hemodialysis.

**Table 3.** cTTP Treatment Patterns

**Treatments administered during acute TTP events^a^ (N = 52)**	**n (%)**
Treatment types received per acute TTP event	
1	32 (61.5)
2	18 (34.6)
3	7 (13.5)
Types of treatments received during acute TTP events	
Plasma exchange	30 (57.7)
Fresh frozen plasma	25 (48.1)
Hemodialysis	1 (1.9)
Other	31 (59.6)
**Treatments received during regular prophylaxis in the post hoc analysis^a,b^ (N = 25)**	**n (%)**
Types of prophylaxis treatment	
Fresh frozen plasma	24 (96.0)
FVIII products	2 (8.0)
Prophylaxis treatment frequency	
Once weekly	4 (16.0)
Every other week, every 14 days, or every 15 days	14 (56.0)
Every 3 weeks, or every 21 days	5 (20.0)
Once per month	2 (8.0)
Other^c^	4 (16.0)

Abbreviations: cTTP, congenital thrombotic thrombocytopenic purpura; FVIII, factor VIII; TTP, thrombotic thrombocytopenic purpura.

^a^N and n refer to the number of patients. Patients can have more than 1 type of treatment and/or more than 1 frequency. Percentages do not necessarily add to 100.

^b^The post hoc analysis focused on the subset of patients who had similar characteristics to those who received plasma-based therapy prophylaxis in a phase 3 pivotal trial (NCT03393975).

^c^Other frequencies (all n = 1) were every 22 days, every 3 weeks then 2 then 3, every 2-3 weeks, and bimonthly since January 2005.

### Post Hoc Analysis

Of the 78 patients included in the study, 25 (32.1%) met criteria similar to the patients who received PBT prophylaxis in the phase 3 pivotal trial (see **Supplementary Methods** and **Supplementary Figure S2**). Of these 25 patients, 17 (68.0%) were female and the median (range) age at study index was 23.0 (1-67) years (**Table 1**). The majority of patients (n = 16, 64.0%) were diagnosed in childhood, with the remaining 9 (36.0%) patients diagnosed in adulthood outside of pregnancy. Patients contributed to a total of 164.4 person-years of follow-up while receiving regular prophylaxis. In this post hoc analysis, data from prophylaxis use during pregnancy were excluded, consistent with this exclusion criterion in the phase 3 pivotal trial.

Nearly all (n = 24, 96.0%) of these patients received FFP, while 2 (8.0%) patients received plasma-derived factor VIII-VWF concentrates (including 1 patient who received both; **Table 3**). The majority of the patients received prophylaxis every week (n = 4, 16.0%) or every other week (n = 14, 56.0%). The median (range) duration of prophylaxis use was 6.3 (0.3-12) years. Clinical symptomatology was the most common reason for starting prophylaxis (n = 10, 40.0%), followed by the frequency of acute TTP events (n = 7, 28.0%). During prophylaxis, 18 (52.9%) treatments were interrupted or stopped for reasons including complications or adverse events, suboptimal clinical benefits, or switching to different regimens.

In total, 4 (16.0%) patients experienced 9 acute TTP events while receiving regular prophylaxis, yielding a rate of 0.0548 events PPY. Infection was the precipitating factor for 5 of these events, with no precipitating factor recorded for the remaining 4 events. Among the 9 acute TTP events, gastrointestinal-related symptoms were the most common presenting symptom (5 events), followed by systemic manifestations (3 events). Purpura/bruising/petechiae due to thrombocytopenia was recorded for 2 events.

## DISCUSSION

This large, multinational, retrospective cohort study demonstrated the substantial disease and treatment burden for individuals with cTTP. Despite the hereditary nature of this condition, nearly three-quarters of patients were not diagnosed until adulthood, including 35% diagnosed in adulthood outside of pregnancy. These patterns of delayed diagnosis reflect those observed previously in registry-based studies of patients with cTTP in the United Kingdom and France.^6,18^ Given that diagnosis often results in the initiation of prophylactic therapy, late recognition of cTTP may lead to poor long-term health outcomes such as end-organ damage, as observed in this study.^6^

Acute TTP events affected the majority of our cohort during follow-up. The incidence of acute TTP events appeared lower during periods of prophylaxis than during periods without prophylaxis, although no statistical assessment was performed (0.050 and 0.202 events PPY, respectively). Although the magnitude of this difference may appear small, small absolute differences in acute TTP event rates can have significant benefit, as these events may be associated with organ damage and a high burden on patients’ overall health. More modest trends were observed by the International Hereditary TTP Registry, which reported rates of 0.36 vs 0.41 events PPY with vs without prophylaxis, respectively, in patient data from August 2018 to December 2019.^8^ However, a more recent, longer-term analysis of that registry (2006 to December 2021) revealed a much more pronounced difference, with Schraner et al reporting that the incidence of acute TTP events was approximately halved during periods of prophylaxis exposure (0.272 [95% CI, 0.231-0.319] PPY) vs non-exposure (0.520 [0.402-0.663] PPY).^19^ Similarly, in an analysis of the French Thrombotic Microangiopathy Registry, prophylaxis was associated with a marked improvement in relapse-free survival among patients with cTTP.^18^ Differences in absolute rates of acute TTP events between studies may be due to differences in how acute TTP events are defined. However, results from multiple studies suggest a role of prophylaxis in reducing the risk of acute TTP events.

Clinical trials have similarly reported on the rate of acute TTP events during regular PBT prophylaxis. When controlling for patient characteristics and definition of an acute TTP event in the post hoc analysis of the present study, the acute TTP ER appeared similar between patients receiving regular PBT prophylaxis in this study (0.0548 events PPY) and the ER observed in patients in the standard therapy arm in a phase 3 clinical trial (0.05 events PPY).^16^ Although these data are consistent with reduced acute TTP event rates during periods of prophylaxis, these rates were not statistically compared with periods without prophylaxis.

The clinical consequences of acute TTP events recorded in this cohort study were medically significant and systemic, including organ damage and sometimes death. The most common types of organ damage were neurological, renal, and cardiac. Importantly, no acute TTP events resulted in organ damage among the subgroup of patients receiving regular prophylaxis in the post hoc analysis. In a Japanese cTTP registry-based study, renal impairment, followed by stroke, were the most common types of long-term organ damage among patients with cTTP, with cardiac hypofunction also reported.^20^ Other studies have reported rates of 25% to 31% for ischemic stroke/transient ischemic attack in patients with cTTP,^6,7,9^ often occurring at early ages.^7,21,22^ Findings on long-term organ damage should be interpreted with caution due to the small number of patients who underwent annual organ assessments during follow-up.

Depression was the most common comorbidity, consistent with prior reports of a high prevalence and impact of depression and other mental health issues among people with cTTP.^18,21,23^ Considering that these data were based on medical records primarily from hematology specialist centers, without the aid of quality-of-life or other validated instruments for collecting patient-reported outcomes, it is likely that the prevalence of depression and other mental health issues were underestimated. Reasons for the high prevalence of mental health issues among patients with cTTP were not ascertained in this study. Possible reasons could be a high psychosocial burden of cTTP, which may result from the deleterious effects of acute TTP events, or the treatment burden of lifelong, regularly administered prophylactic infusions. As pregnancy is a common precipitating factor for acute TTP events,^9^ the disease burden patients experience may be particularly compounded during this vulnerable period. These findings highlight a need for more research on the impact of cTTP on mental health and quality of life.

Over one-third of patients had TTP manifestations, the most common being thrombocytopenia. This finding reflects the central role of platelet consumption and platelet-rich microthrombi in the pathophysiology of cTTP.^1,2^ The most common symptoms observed in this study were non-overt, such as abdominal pain, lethargy, and headaches. Combined data from the UK TTP Registry and the South East England Registry for TTP suggest that non-overt symptoms do not necessarily occur alongside clinical and laboratory features of TTP.^6^ These prior registry data are consistent with lower acute TTP event rates and improved outcomes during periods of prophylaxis compared with periods without prophylaxis, although these observational comparisons remain vulnerable to confounding and selection bias.^6^ Although not evaluated in the present study, the observed rates of acute TTP events and TTP manifestations, and the high prevalence of comorbidities are expected to be associated with high economic burden and impacts on quality of life.

In the post hoc analysis of patients receiving regular prophylaxis, most patients received PBT prophylaxis every 1 or 2 weeks, similar to prior registry studies.^6,9,20^ However, most treatments were stopped or interrupted, largely due to complications or suboptimal clinical benefit. This is consistent with a prior real-world study in which 35% of patients receiving FFP prophylaxis experienced treatment-related adverse events.^18^ PBTs are known to have several treatment limitations. They must be administered in a clinical setting and are associated with large infusion volumes that are time-consuming to administer and may cause volume overload, particularly in patients with cardiac or kidney impairment.^2,13^ PBTs also carry a risk of allergic reactions, including anaphylaxis, and potential pathogen transmission.^2,20,24^ Although premedication with anti-allergic agents and steroids can reduce the risk of allergic reactions, some patients still experience them despite premedication.^20,24^ This suggests a need for alternative ADAMTS13 replacement therapies that can more effectively address the underlying ADAMTS13 deficiency for both short- and long-term disease control.

At the time of data collection, PBT treatment was considered standard of care. However, guidelines published in 2025 by the International Society on Thrombosis and Haemostasis recommend that patients with cTTP receive prophylaxis to prevent acute TTP events and minimize symptom burden, with recombinant ADAMTS13 (rADAMTS13) recommended over PBT to prevent acute TTP events for patients who are in remission.^25,26^ This recommendation was based on interim data from a phase 3 clinical trial in which rADAMTS13 provided higher peak levels and higher overall ADAMTS13 activity exposure compared with PBTs.^16^ Future developments may include specific recommendations for pediatric patients, and further research on the long-term effectiveness of rADAMTS13, particularly with respect to disease-related long-term organ damage, and treatment and healthcare burden over the course of a lifetime.

Strengths of this observational study include the substantial sample size relative to the rare cTTP population and the long follow-up period. However, this study has limitations, including potential bias, missing data, and confounding factors. Firstly, participants were selected based on convenience sampling, and most participating sites were academic centers. Therefore, patients may have more severe disease courses with more organ damage compared with the general cTTP population. Immortal time bias could also have arisen because some patients commenced prophylaxis after the study was initiated, limiting the time period during which adverse outcomes could have occurred. Information bias may have resulted from the broad definition of index events, which could increase heterogeneity in baseline disease risk. Secondly, the retrospective, noninterventional study design impacts data completeness, as raw data captured by sites were not prospectively determined or mandated. A third key limitation is the potential for confounding. No adjustment was made for confounders or time-varying exposures (such as prophylaxis), which would limit causal interpretation. There was also substantial missing data on race/ethnicity due to country-specific restrictions on collecting ethnicity data in France. As inferential analyses were not conducted, formal comparisons could not be made between subgroups, including comparing periods of prophylaxis to periods without prophylaxis. Also, the post hoc analysis was conducted on a subset of patients meeting the entry requirements of the cTTP rADAMTS13 phase 3 trial^16^; therefore, the results cannot be directly compared with the overall cohort. Finally, the data span of 2009 to 2020 may limit generalizability to the present day.

Most patients in this study were from France and treated according to the standards recommended by the French Reference Center for Thrombotic Microangiopathies (CNR-MAT). Therefore, patients treated according to these recommendations are expected to be broadly representative of patients with cTTP across the whole of France.^27^ However, treatment practices in France may differ from other countries, as France has a centralized model of care with structured longitudinal follow-up, and historically high uptake of prophylaxis, particularly during high-risk periods such as pregnancy.^18,27,28^ This could have led to a higher than expected rate of prophylaxis use and possibly lower rates of acute TTP events, TTP manifestations, and organ damage, compared with less-treated populations in other geographies. It should also be noted that all the participants in this study were in Europe or the United States, and generalizability to countries outside these regions may be limited. Therefore, additional research to assess the global burden of cTTP is warranted. Studies using medical information from multinational populations could further illustrate the ways in which cTTP impacts patients and health systems.

In conclusion, in this multinational, retrospective, longitudinal cohort, individuals with cTTP experienced a sustained and substantial burden of disease and treatment despite long-term plasma-based prophylaxis. Our findings are consistent with lower observed acute TTP event rates during periods of PBT prophylaxis, while also highlighting the limitations of PBT in preventing ongoing manifestations, cumulative organ damage, and the logistical and safety burdens associated with repeated plasma infusions. Overall, this study suggests that more effective treatments may be needed.

### Disclosures

P.C. has received speaker fees and advisory board honoraria from Alexion, Janssen, Novartis, Sanofi, and Takeda. M.S. has received speaker fees and advisory board honoraria from Octapharma, Sanofi, and Takeda, and research grants from Alexion, Baxalta (a Takeda company), and Shire (a Takeda company). J.A.K.H. has received speaker fees and advisory board honoraria from Sanofi and Takeda, and research grants from Baxalta (a Takeda company). B.S.J. is a member of the French advisory boards for Alexion and has received speaker fees from Sanofi and Takeda. A.V. is a member of the French Advisory boards for caplacizumab (Sanofi) and recombinant ADAMTS13 (Takeda). P.D. and B.M. are employees of Takeda Development Center Americas, Inc., and were stockholders of Takeda at time of study. P.P., L.T.W., H.F., and R.S. are employees of Takeda Development Center Americas, Inc., and stockholders of Takeda.

### Ethics Approval and Consent to Participate

The study was conducted in accordance with the Declaration of Helsinki, and followed the Guidelines for Good Pharmacoepidemiology Practices, recommended by the International Society for Pharmacoepidemiology. Institutional review board/independent ethics committee approval was obtained from each participating study site as required by local regulations. Written informed consent or a waiver of informed consent was obtained as applicable and in accordance with the requirements of the relevant ethical committees in each country.

## Supplementary Material

Online Supplementary Material

## Data Availability

Data sets supporting the results from this study are available from the corresponding author upon reasonable request. The data sets will be provided after de-identification, in compliance with applicable privacy laws, data protection, and requirements for consent and anonymization.
