# Supplementary material for: Natural History and Treatment Outcomes of Congenital Thrombotic Thrombocytopenic Purpura: A Retrospective Longitudinal Cohort Study
Source: J Health Econ Outcomes Res. 2026 Aug 12;13(2):55–66. doi: 10.36469/001c.164543 (PMC13476067; doi:10.36469/001c.164543)
Supplement: Supplementary file 1 [file jheor_2026_13_2_164543_358522.pdf]

Journal of Health Economics and Outcomes Research

Online Supplementary Material

Natural History and Treatment Outcomes of Congenital Thrombotic Thrombocytopenic Purpura: A Retrospective Longitudinal Cohort Study. *JHEOR*. 2026;13(2):55-66. doi:10.36469/jheor.2026.164543

Statistical Analysis Plan **1**

Supplementary Materials **30**

STROBE Checklist **39**

This supplementary material has been provided by the authors to give readers additional information about their work.

This is an open-access article distributed under the terms of the Creative Commons Attribution 4.0 International License (CCBY-4.0). View this license's legal deed at http://creativecommons.org/licenses/by/4.0 and legal code at http://creativecommons.org/licenses/by/4.0/legalcode for more information.

NON-INTERVENTIONAL (OBSERVATIONAL) STATISTICAL ANALYSIS PLAN

Title: *Natural History and Treatment Outcomes of Congenital Thrombotic Thrombocytopenic Purpura: A Retrospective Chart Review Study*

Protocol Short Title: *Retrospective Chart Review of TTP in EU4 (France, Germany, Italy, Spain), UK, Switzerland, USA, and Japan*

Protocol Version and Date TAK-755-3003, Version 1.4 11th April 2022

Registration No.: *Study not registered*

Active Substance: *Not applicable*

Medicinal Product: *Not applicable*

Product Reference: *Not applicable*

Sponsor/MAH: *Takeda*

Country(-ies) of Study: *EU4 (France, Germany, Italy, Spain), UK, Switzerland, USA, and Japan*

Takeda Study Lead: *Ragy Saad, Director, Global Evidence and Outcomes*

This document contains confidential and proprietary information of Takeda and is disclosed pursuant to confidentiality and non-disclosure obligations. This information should be used solely for the purposes for which it was provided and should not be copied, shared with, or disclosed to any third party without the express written consent of Takeda.

Takeda Pharmaceuticals International, Inc.

TABLE OF CONTENTS

TABLE OF CONTENTS 2

LIST OF TABLES 3

LIST OF FIGURES 3

ABBREVIATIONS 4

1 INTRODUCTION 6

2 RESEARCH QUESTION AND OBJECTIVES 6

2.1 Research Questions 6
2.2 Objectives 6
2.2.1 Primary objectives 7
2.2.2 Secondary objectives 7
2.2.3 Exploratory objective 7

3 RESEARCH METHODS 7

3.1 Study Design 7
3.1.1 Index event definitions 9
3.2 Study Setting and Study Population 10
3.2.1 Number and Type of Patients 10
3.2.2 Sites and Regions 10
3.3 Patient Eligibility Criteria 10
3.3.1 Inclusion Criteria 10
3.3.2 Exclusion Criteria 11
3.3.3 Discontinuation of Participants 11
3.4 Data Source/Data Collection: 11
3.4.1 Data Security 12
3.5 Variables of Interest 12
3.5.1 Primary Outcomes 12
3.5.2 Other study variables (characteristics of cTTP patients) 13
3.5.3 Secondary Outcomes 17
3.6 Data Analysis 22
3.6.1 General statistics 22
3.6.2 Analysis of primary outcomes 23
3.6.3 Analysis of secondary outcomes 23

3.7 Data Management 24
3.7.1 Missing data 24
3.7.2 Data cleaning 24
3.7.3 Quality control 24

REFERENCES 26

APPENDIX 27

LIST OF TABLES

Table 1: Index events and patient observability 13
Table 2: Sociodemographic characteristics 14
Table 3: Clinical characteristics 15
Table 4: Characteristics at cTTP diagnosis 16
Table 5: Pregnancy and Pregnancy-Related Outcomes (Female patients older than 14 years of age at study index) 16
Table 6: Treatment at acute events during the study period 18
Table 7: Treatment at isolated TTP manifestation at index only 19
Table 8: Prophylaxis treatment during the study period 20
Table 9: Patient's HRU 21
Table 10: Precipitating factors 27
Table 11: Treatments 27
Table 12: Treatment related complications (for PEX, FFP and CSP only) 27
Table 13: Treatment frequency 27
Table 14: Specialties 28
Table 15: Comorbidities 28
Table 16: Symptoms 28
Table 17: Pregnancy-related complications 29

LIST OF FIGURES

Figure 1: Study Design Patient Flow Diagram 8

ABBREVIATIONS

ADAMTS13	A disintegrin and metalloproteinase with a thrombospondin type 1 motif, member 13
AOA	Annual organ assessment
BMI	Body mass index
cTTP	Congenital Thrombotic thrombocytopenic purpura
CRF	Case Report Form
CRO	Contract Research Organization
CSP	Cryosupernatant plasma
CVC	Central vein catheter
DIC	Disseminated intravascular coagulation
ECG	Electrocardiogram
eCRF	Electronic case report form
ER	Event rate
FFP	Fresh frozen plasma
GU	Genitourinary
HIV	Human Immunodeficiency Virus
HUS	Hemolytic Uremic Syndrome
HRU	Healthcare resource utilization
ICU	Intensive care unit
IDA	Iron deficiency anemia
IQR	Interquartile range
PEX	Plasma exchange
PLT	Platelet
SAP	Statistical analysis plan
SD	Standard deviation
SDP	Solvent/detergent-treated plasma
SE	Standard error
SLE	Systemic Lupus Erythematosus
SoC	Standard of care
ST/T	ST-segment and T-wave

TIA	Transient ischemic attack
TTP	Thrombotic thrombocytopenic purpura
ULN	Upper limit of normal

1 INTRODUCTION

The purpose of this statistical analysis plan (SAP) is to provide details on the statistical analyses that have been outlined within the protocol for the *"Natural History and Treatment Outcomes of Congenital and Immune Thrombotic Thrombocytopenic Purpura: A Retrospective Chart Review Study"* (TAK-755-3003). The scope of this analysis plan is for congenital Thrombotic Thrombocytopenic Purpura (cTTP) only and includes the description of the study population, collection of data and all prespecified analyses to be executed by HCD economics. The analyses to be performed include a descriptive analysis, primary and secondary objective analyses, and an exploratory analysis.

2 RESEARCH QUESTION AND OBJECTIVES

2.1 Research Questions

- What are the short- and long-term disease related outcomes, signs and symptoms, burden of disease, presence of organ damage, survival, therapeutic management, treatment patterns, resource utilization and natural history of patients with cTTP?
- How are cTTP clinically managed in a real-world care setting?
- What is the burden associated with long term complications (neurological, renal and cardiac) of cTTP?
- Is there a correlation between the patients' clinical status and laboratory markers such as ADAMTS13, LDH, platelet count (pre/during/post-acute event), hemoglobin levels, ADAMTS13 autoantibodies, etc., or any clinical or sub-clinical disease manifestations (such as acute events or isolated TTP manifestations)?
- Is there a correlation between ADAMTS13 or other biomarker levels and short- and long-term outcomes; do these biomarkers hold prognostic value?

2.2 Objectives

The aim of this study is to describe the natural history of patients with cTTP, characterize short- and long-term outcomes, burden of disease, and survival, as well as to examine treatment patterns, and healthcare resource utilization. Additionally, the study will explore whether a link can be established between ADAMTS13 and other biomarker levels, and short- and long-term outcomes to assess their prognostic value.

2.2.1 Primary objectives

The primary objective of the study is to describe the natural history of disease in cTTP including:

- Prevalence and incidence[1] of short- and long-term clinical manifestations of cTTP
- Prevalence and incidence[2] of short- and long-term disease-related complications
- Characteristics of cTTP patients

2.2.2 Secondary objectives

The secondary objectives of the study are to:

- Describe treatment patterns and treatment-related outcomes
- Describe healthcare resource utilization

2.2.3 Exploratory objective

The exploratory objectives of the study are to:

- Describe the relationship between ADAMTS13 activity level and markers of organ damage
- Describe the relationship between ADAMTS13 activity level and acute, isolated, chronic/long-term progressive complications and death.

3 RESEARCH METHODS

3.1 Study Design

This study is a non-interventional, retrospective chart review. The source of all data collected will be the medical records/charts of patients with confirmed diagnosis of cTTP and the investigators will be specialist physicians with experience of treating thrombotic microangiopathies. The study outcomes are designed to address the key objectives

[1] There was limited collection of data outside the study period, therefore it was not possible to calculate incidence as it was not possible to clearly identify when events were occurring for the first time.

[2] See footnote 1.

of the study including patient characteristics, short- and long-term clinical manifestations and disease related complications, and healthcare resource utilization.

This study meets the criteria of a retrospective study defined in Directive 2001/20/EC and followed the guidelines for good pharmacoepidemiology practices (GPP) recommended by International Society for Pharmacoepidemiology (1). This indicates that:

- The choice of a particular therapeutic strategy to a patient is not decided in advance by the study protocol but fell within current practice.
- No additional diagnostic or monitoring procedures are applied to the subjects.

Confirmed cTTP patients will be included into the study based on the index event that happened between January 1st, 2009, to December 31st, 2017, and the study period will be from January 1st, 2009, to death, loss to follow up in care, enrollment in a clinical trial, or December 31st, 2020, whichever occurred first. For patients who are involved or are enrolled at the time of data collection in a clinical trial, only data generated before enrollment in the clinical trial will be abstracted, i.e., sites will be requested to not include data from the date that patients enrolled into clinical trials. The execution of this study will be from December 2020 until March 2024, with data abstraction taking place between February 2021 and December 2022. The study design is shown in **Figure 1**.

Figure 1: Study Design Patient Flow Diagram

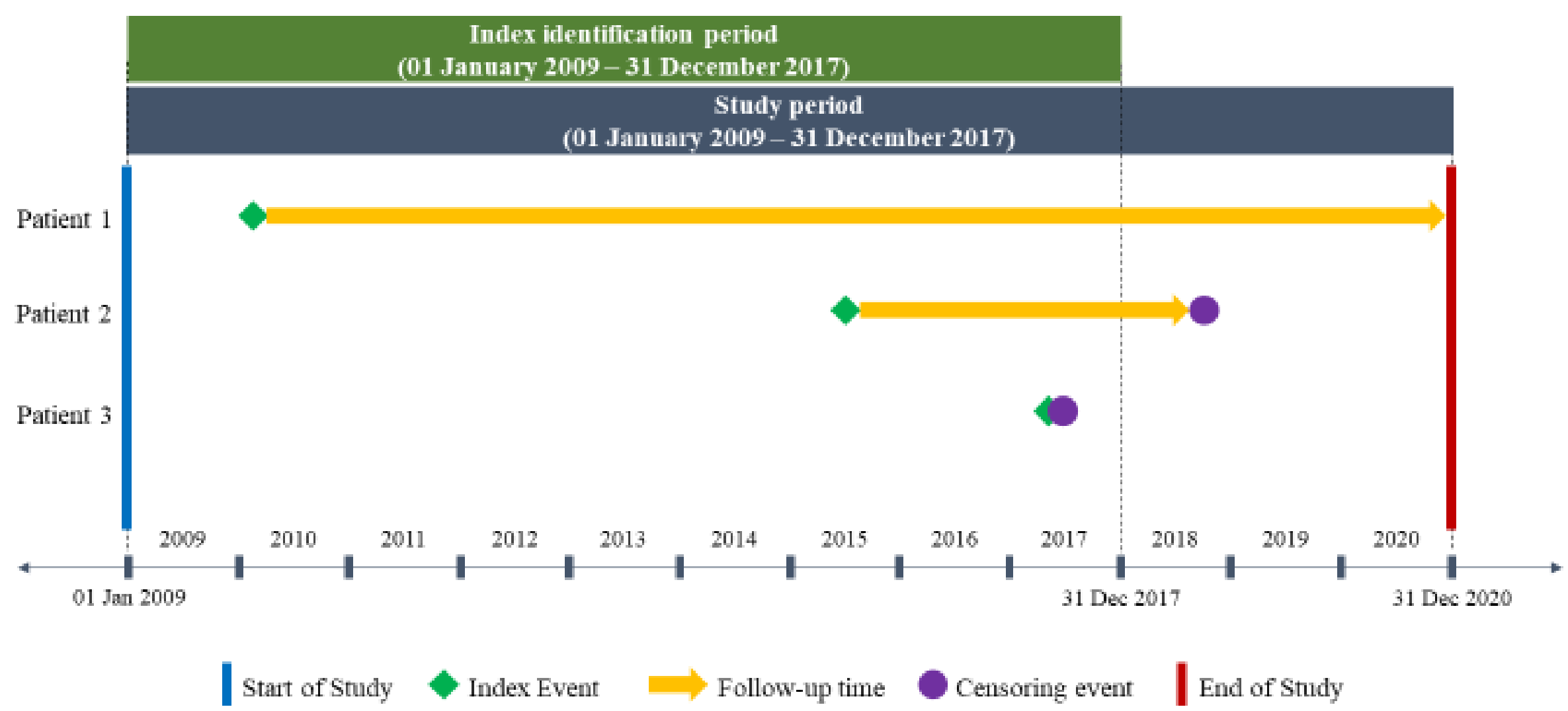

Patients 1, 2, and 3 are hypothetical patients.
Possible censoring events: loss to follow-up, enrollment in a clinical trial, death.

3.1.1 Index event definitions

- Acute TTP events (in the eCRF, called 'acute episodes') will be defined as the presence of both of the following criteria[3]
    - Thrombocytopenia: drop in platelet count ≥ 50% of baseline OR a platelet count < 100,000/μL
    - Microangiopathic hemolytic anemia: elevation of LDH > 2xupper limit of normal (ULN)

- Isolated TTP manifestations (in the eCRF, called 'subacute manifestations') will be defined based on the presence of any of the following criteria[4]:
    - Thrombocytopenia: drop in platelet count ≥ 25% of baseline at screening OR a platelet count < 150,000/μL
    - Microangiopathic hemolytic anemia: elevation of LDH>1.5xULN
    - Signs and symptoms including:
        - Other neurological symptoms (e.g., confusion, memory issues, irritability, dysarthria, focal or general motor symptoms including seizures, headache) as per the opinion of the investigator/treating physician.
        - Lethargy
        - Abdominal pain
        - Increase in serum creatinine >1.5xbaseline

- TTP diagnosis will be used if the patient was first diagnosed with TTP within the index period.

- Prevention of TTP will be defined as the administration of prophylactic treatment.

- TTP-related clinical event will be defined as a cerebrovascular event, cardiovascular event, thrombosis, organ-related event, GI event, bleeding, neurologic event, or any other acute clinical event that is related to TTP.

[3] Clinical trial.gov: A Study of BAX 930 in Children, Teenagers, and Adults Born With Thrombotic Thrombocytopenic Purpura (TTP) - Tabular View. (ClinicalTrials.gov identifier: NCT03393975).

[4] Clinical trial.gov: A Study of BAX 930 in Children, Teenagers, and Adults Born With Thrombotic Thrombocytopenic Purpura (TTP) - Tabular View - ClinicalTrials.gov

3.2 Study Setting and Study Population

This study is a multi-center retrospective chart review conducted in France, Germany, Italy, Spain, Switzerland, Japan, the United Kingdom (UK), and the United States (US). Data will be collected from electronic medical records at sites where the patients will be identified.

3.2.1 Number and Type of Patients

Given the rarity of the disease, the intention for this study is to be inclusive of as many patients as possible with a confirmed diagnosis of cTTP (2-4). Convenience sampling from the target population (patients with a confirmed diagnosis of cTTP at participating sites) will be employed to achieve the target patient number (N=80). No other sampling method will be used.

3.2.2 Sites and Regions

Institutions in the EU4 countries (France, Germany, Italy, and Spain), Japan, Switzerland, the United Kingdom (UK), and the United States (US) will be invited to participate in the study.[5] Sites will be selected based on physicians' experience in treating TTP and the number of eligible patients at each site. Sites will also be evaluated for their abilities to conduct research and will be selected if they had previous research experience and are able to conduct retrospective chart reviews.

3.3 Patient Eligibility Criteria

3.3.1 Inclusion Criteria

Patient eligibility will be determined according to the following criteria:

- Subject has a documented diagnosis of severe hereditary ADAMTS13 deficiency, defined as:
    - Confirmed by molecular genetic testing, documented in subject history or at screening or

[5] Sites in Austria and Norway were also invited, but did not participate in the study.

- A severe ADAMTS13 deficiency (ADAMTS13 activity ≤10% of normal) in the absence of an ADAMTS13 inhibitor, documented in plasma samples withdrawn at two different time points with an interval of more than 14 days (subjects currently receiving SoC prophylactic therapy may exceed 10% ADAMTS13 activity at screening)

and/or

- Confirmed cTTP diagnosis based on other criteria specified by the healthcare professional (locally used criteria)

3.3.2 Exclusion Criteria

The only exclusion criterion will be a confirmed diagnosis of any other hematological disorder (with the exception of Hemolytic Uremic Syndrome [HUS]) (5-9).

3.3.3 Discontinuation of Participants

Not applicable, because of the retrospective nature of the study.

3.4 Data Source/Data Collection:

This study will be a multi-center retrospective chart review conducted in selected countries. Data will be collected from electronic medical records of eligible patients with cTTP. Paper records will be used where electronic records are unavailable. Existing data will be extracted from patient medical records and entered into an electronic Case Report Form (eCRF). The eCRF will be built and hosted on a platform provided by the vendor, CISIV Ltd. This vendor will be responsible for ensuring that the eCRF will be hosted as per the CRO's requirements in order to be compliant with the study protocol. The eCRF will not collect or have any links to identifiable information or protected health information, such as name, address, date of birth or social security number. A single eCRF will be completed for each included patient. A propriety dataset of data from the CRFs will be created in order to complete the data analyses. No other data will be required for this study.

Study variables to be extracted included relevant clinical outcomes, demographic characteristics, and information on healthcare resource utilization (see **Section 3.5**).

3.4.1 Data Security

The CRO will provide security measures against unauthorized access to data. The security measures will include:

- User Security: Users logging into the system will have level-specific access to information based upon assigned rights.
- Network Security: Users will be required to log into the network before accessing any information.
- Laptop security: All laptops will be encrypted.
- Database Security: Our databases will provide security features that permitted users to access only the information that is relevant to their position, including encrypted passwords, internal and external user authentication, IP address restrictions, fine-grained database privileges, and group level access control.
- Materials: All study materials will reside in restricted-access areas of our networks. Only specific project staff will have access to these folders.
- Building Security: All buildings will be secure and require fob access at all times with security on reception.

3.5 Variables of Interest

3.5.1 Primary Outcomes

This section describes the primary outcomes to be collected to address the primary study objectives. For a description of the planned analyses for each outcome, please see **Section 3.6.2**.

Primary objective 1: Prevalence/ incidence of short and long-term clinical manifestations of cTTP.

1. Prevalence and event rate of acute TTP events (continuous)
2. Proportion of patients with acute events: Patients with 0 events, 1 event, 2 events, 3 events, 4 events or 5 or more events (continuous)
3. Clinical presentation (symptoms) at acute events (categorical)
4. Precipitating factors for acute TTP events (categorical: yes, no)
    a. If yes, select precipitating factor: Please see **Table 10** in the Appendix for list of precipitating factors.

5. Clinical outcomes of acute events: Outcome of event (resolved / not resolved); time to resolution; complete ADAMTS13 remission;[6] time to ADAMTS13 remission; PLT count normalization; time to PLT count normalization
6. Prevalence and event rate of isolated TTP manifestations (continuous)
7. Clinical presentation (symptoms) at isolated TTP manifestations (categorical)
8. Clinical outcomes of isolated TTP manifestations: Outcome of event (resolved / not resolved); time to resolution.

Primary objective 2: Prevalence/incidence of short and long-term disease-related complications.

1. Organ Damage: based on physician's assessment
    a. Prevalence and event rate of any organ damage at acute events (continuous). Prevalence will also be reported by type of organ damage (cardiac, neurological, renal, liver).
    b. Prevalence of organ dysfunction at annual organ assessments (ST/T waves changes, ischemia, liver damage: "was liver damage detected?") and order detected
2. Death
    a. Death (categorical): "Is the patient alive" (when completing CRF)? Yes, No.
    b. Cause of death (categorical): TTP treatment-related; TTP event-related; TTP-related major clinical event; Not TTP-related; Unknown.

3.5.2 Other study variables (characteristics of cTTP patients)

The following variables will be described for all patients, at different timepoints: at diagnosis, at index event, during the entire disease course (at any point in the patient's medical history including the pre-study period).

Table 1: Index events and patient observability

Variable	Variable definition	Type	Timepoint	Categories (if type = categorical)
Follow up time (years)	The number of years in the study period	Continuous	Study Period	

6 In the eCRF, called 'ADAMTS13 normalization (ADAMTS13>50%)'.

Variable	Variable definition	Type	Timepoint	Categories (if type = categorical)
Index event	Type of index event	Categorical	Index	• TTP diagnosis<br>○ Diagnosis corresponds to acute event<br>○ Diagnosis did not correspond to acute event<br>• TTP acute event<br>• Isolated TTP manifestation<br>• Prophylactic treatment for TTP<br>• TTP-related major clinical event

Table 2: Sociodemographic characteristics

Variable	Variable definition	Type	Timepoint	Categories (if type = categorical)
Sex	Patient sex	Categorical	Index	• Male<br>• Female
Age at index date	Patient age (years)	Continuous	Index	
Age at index date	Patient age (years)	Categorical	Index	• Under 18<br>• 18-29<br>• 30-44<br>• 45-64<br>• ≥65
Ethnicity	Patient's ethnicity	Categorical	Index	• White/Caucasian<br>• Black/Afro-Caribbean<br>• Middle Eastern<br>• Asian-Indian subcontinent<br>• Asian-other<br>• Hispanic/Latino<br>• Prefer not to answer

Variable	Variable definition	Type	Timepoint	Categories (if type = categorical)
Country	Patient's country of origin	Categorical	Index	• France<br>• Germany<br>• Italy<br>• Spain<br>• UK<br>• Switzerland<br>• Japan<br>• USA

Table 3: Clinical characteristics

Variable	Variable definition	Type	Timepoint	Categories (if type = categorical)
BMI	Patient's BMI	Categorical	Index or closest available date	Adults (≥20 years old) will be categorized as:<br>• underweight (BMI<18.5),<br>• normal weight (18.5≤BMI<25)<br>• overweight (25≤BMI<30)<br>• obese (BMI≥30)
Smoking Status	Patient's smoking status	Categorical	Up to the date of data entry	• Yes, currently smokes<br>• Yes, used to smoke and now stopped<br>• No, never smoked<br>• Unknown
Alcohol dependence	Whether patient has experienced alcohol dependence	Categorical	Up to the date of data entry	• Yes<br>• No
Drug dependence	Whether patient has experienced drug dependence	Categorical	Up to the date of data entry	• Yes<br>• No
Comorbidities	Patient's comorbidities	Categorical	Up to the date of data entry	See **Table 15** in the Appendix for a list of comorbidities.

Table 4: Characteristics at cTTP diagnosis

Variable	Variable definition	Type	Timepoint	Categories (if type = categorical)
Patient's age at diagnosis	Calculated using the age at index (see **Table 2**) and the quarters of index and of diagnosis. $Age_{diag} = Age_{index}$ - (index quarter – diagnosis quarter)/4	Continuous	Diagnosis	
Diagnosis basis	Patient's basis for TTP diagnosis	Categorical	Diagnosis	• Severe ADAMTS13 deficiency (<10%)<br>• Family History<br>• Genetic sequencing
Family history of cTTP	Patient's family history of cTTP	Categorical	Index	• Father<br>• Mother<br>• Siblings<br>• Other (paternal)<br>• Other (maternal)
Clinical presentation (symptoms) at diagnosis	Symptoms patient experienced at diagnosis	Categorical	Diagnosis	See **Table 16** in the Appendix for list of symptoms

Table 5: Pregnancy and Pregnancy-Related Outcomes (Female patients older than 14 years of age at study index)

Variable	Variable definition	Type	Timepoint	Categories (if type = categorical)
TTP diagnosis at pregnancy	Was the patient diagnosed during or immediately following pregnancy	Categorical	Diagnosis	• Yes<br>• No<br>• Unknown

Variable	Variable definition	Type	Timepoint	Categories (if type = categorical)
Pregnancy history	If patient has ever been pregnant	Categorical	Up to the date of data entry	• Yes – one time<br>• Yes – two times<br>• Yes – three times<br>• Yes – more than three times<br>• No<br>• Unknown
Pregnancy-related complications	Patient's pregnancy related complications	Categorical	Up to the date of data entry	See **Table 17** in the Appendix for pregnancy complications
Pregnancy-related complications at acute events	Pregnancy related complications at acute events	Categorical	Acute events during the study period	See **Table 17** in the Appendix for pregnancy complications
Acute event treatment, if pregnant at time of the event	TTP treatment administered	Categorical	At acute events during the study period	See **Table 11** in the Appendix for list of treatments at acute events

3.5.3 Secondary Outcomes

This section describes the secondary outcomes collected to address the secondary study objectives. For a description of the planned analyses for each outcome, please see **Section 3.6.3**.

Secondary Objective 1: Treatment patterns and treatment-related outcomes
Treatments (and outcomes if applicable) will be reported for: (1) treatment at acute events (during whole study period) (2) treatment at isolated TTP manifestations (at index only) and (3) prophylaxis treatment during whole study period.

1. Treatment for acute events

Table 6 shows the outcomes relating to treatment of acute events during the study period which will be reported.

Table 6: Treatment at acute events during the study period

Variable	Variable definition	Type	Timepoint	Categories (if type = categorical)
TTP treatment	Number of types of treatments used	Continuous	At acute events during the Study period	
TTP Treatment	TTP treatment administered	Categorical	At acute events during the Study period	See Table 11 in the Appendix for list of treatments
CVC insertion by event	Was CVC insertion necessary?	Categorical	At acute events during the Study period	• Yes<br>• No<br>• No, already present
Outcome of event	Whether the acute event resolved	Categorical	At acute events during the Study period	• Resolved, no complications<br>• Resolved, short term complications<br>• Resolved, organ damage<br>• Not resolved – patient deceased
Time to resolution from treatment initiation or from onset of an acute event?	How fast the event resolved	Categorical	At acute events during the Study Period	• up to 7 days<br>• 8-14 days<br>• >14 days
ADAMTS13 remission	Was complete ADAMTS13 remission (ADAMTS13>50%) achieved?	Categorical	At acute events during the Study Period	• Yes<br>• No
Time to ADAMTS13 remission	How long did it take for complete ADAMTS13 remission to be achieved	Categorical	At acute events during the Study Period	• up to 7 days<br>• 8-14 days<br>• >14 days
PLT normalization	Did PLT count normalize (PLT > 150.000)?	Categorical	At acute events during the Study Period	• Yes<br>• No

Variable	Variable definition	Type	Timepoint	Categories (if type = categorical)
Time to PLT normalization	How long did it take for PLT to normalize	Categorical	At acute events during the Study Period	• up to 7 days<br>• 8-14 days<br>• >14 days
Treatment related complications	Complications related to treatment	Categorical	At acute events during the Study period	• Yes<br>• No
Treatment related complications (for PEX, FFP and CSP only) by treatment	Complications related to treatment	Categorical	At acute events during the Study period	See **Table 12** in the Appendix for list of complications
Complications related to CVC insertion by event	Complications related to treatment	Categorical	At acute events during the Study period	• Yes<br>• No

2. Treatment for isolated TTP manifestations at index event:

Table 7 shows the outcomes relating to treatment of isolated TTP manifestations at index only which will be reported.

Table 7: Treatment at isolated TTP manifestation at index only

Variable	Variable definition	Type	Timepoint	Categories (if type = categorical)
TTP Treatment	TTP treatment administered	Categorical	Isolated TTP manifestation at index	See **Table 11** in the Appendix for list of treatments
Treatment related complications	Complications related to treatment	Categorical	Isolated TTP manifestation at index	• Yes<br>• No
Treatment related complications (for PEX, FFP and CSP only) by treatment	Complications related to treatment	Categorical	Isolated TTP manifestation at index	See **Table 12** in the Appendix for list of complications

3. Prophylaxis treatment for cTTP:

Table 8 shows the outcomes to be collected related to routine prophylactic or pre-emptive treatment. Prophylaxis or pre-emptive treatment will be reported for the entire study period. If prophylaxis started or ended prior to 2009 or after 2020, only the time on prophylaxis during the study period will be considered. All results will be provided for all prophylaxis treatments and for regular prophylaxis.[7]

Table 8: Prophylaxis treatment during the study period

Variable	Variable definition	Type	Timepoint	Categories (if type = categorical)
Reason for initiating prophylaxis	Recorded reason why prophylaxis started	Categorical	Study period	• Clinical symptomology<br>• TTP events frequency<br>• Isolated TTP manifestation frequency<br>• drop in platelet count<br>• other (free text)
Frequency of administration by treatment	How frequently the treatment was administered	Categorical	Study Period	Categories listed in the eCRF (see **Table 13** in the Appendix) are grouped into:<br>• About every 2 weeks<br>• About every week or more<br>• About every 3 weeks<br>• Longer than every 3 weeks<br>• Other
Prophylaxis Treatment	TTP treatment administered	Categorical	Study Period	See **Table 11** in the Appendix for prophylaxis treatments

[7] Prophylaxis status will be defined for each test using its quarter, depending on the type (if any) of prophylaxis the patient received at the time. Prophylaxis with a frequency between ≥ 1 per month and ≤ 3 per week, excluding steroids, will be classified as regular prophylaxis.

Variable	Variable definition	Type	Timepoint	Categories (if type = categorical)
Interrupt, switch or stop treatment	Was the treatment interrupted, stopped or switched?	Categorical	Study Period	• Yes<br>• No
Treatment Duration	Treatment duration	Continuous	Study Period	
Reason for treatment interruption, switch, stop	What was the reason?	Categorical	Study Period	• Complications<br>• Switch (normalization not achieved)<br>• Normalization of levels<br>• Other
Treatment related complications	Complications related to treatment	Categorical	Index	• Yes<br>• No

Secondary Objective 2: Healthcare resource utilization (HRU)

Table 9 shows the endpoints related to healthcare resource use. These endpoints will be reported for acute events (for the study period) and isolated TTP manifestations (index only) separately. Specialist consultations for managing TTP-related comorbidities during the study period will be described as overall and by yearly average.

Table 9: Patient's HRU

Variable	Variable definition	Type	Timepoint	Categories (if type = categorical)
Hospitalization	Did the patient required a stay in the hospital?	Categorical	Study period (acute events) and Index (isolated TTP manifestations)	• Yes<br>• No
Type of hospitalization	The type of hospitalization	Categorical	Study period (acute events) and Index (isolated TTP manifestations)	• Ward<br>• ICU<br>• Day Case

Variable	Variable definition	Type	Timepoint	Categories (if type = categorical)
Number of days (if inpatient care)	The number of days in hospital (ward and ICU) (days)	Continuous	Study period (acute events) and Index (isolated TTP manifestations)	
Specialties involved	Type of specialties involved in treating acute event or isolated TTP manifestation	Categorical	Study period (acute events) and Index (isolated TTP manifestations)	See list of specialists in **Table 14** in the Appendix.
Specialist consultations (outside of acute events and isolated TTP manifestations)	Number of specialist consultations related to comorbidities caused by TTP	Continuous, for each specialty	Study period	See list of specialists in **Table 14** in the Appendix.

3.6 Data Analysis

3.6.1 General statistics

This study will be primarily descriptive in nature and no hypotheses will be tested.

All the analyses will be conducted using baseline packages for statistical analysis in STATA 17 (StataCorp. 2019. Stata Statistical Software: Release 17. College Station, TX: StataCorp LLC.).

All variables will be summarized using descriptive statistics. Categorical variables will be presented as frequencies and percentages. Continuous variables will be reported as mean, median, standard deviation (SD) or standard error (SE), interquartile range (IQR) and range. If one patient has multiple records, the average for each patient will be calculated before calculating the average across all patients.

3.6.2 Analysis of primary outcomes

Prevalence will be reported for acute events, isolated TTP manifestations, and organ damage. It will be calculated as the number of patients who ever experienced an event divided by the total number of eligible patients. Prevalence will be reported as N (%) or per 100 persons.

For organ dysfunction at annual organ assessments, results will be reported for all AOAs during the study period and by their order (i.e., first AOA, second AOA, third AOA, etc.).

Event rates (ERs) will be calculated for acute events and isolated TTP manifestations during the study period. ERs will be calculated to reflect the annualized rate of an event per person-year (PPY) of follow-up given the varying amount of follow-up times each patient contributed during the study. The ER will be calculated using the following formula:

$$Event\ Rate\ (ER) = \frac{\sum_i Number\ of\ Events_i}{\sum_i Number\ of\ Years_i}; i = patient$$

ERs will be further stratified by the following categories:

- Sex (male, female)
- Age group at index (under 18, 18-29, 30-44, 45-64, 65+)
- Time since diagnosis at index (up to 1 year, more than 1 year and up to 5 years, more than 5 years and up to 10 years, more than 10 years)
- Diagnosis year (2008 or earlier, 2009 or later)
- Prior history of acute events (no acute events before the study period (incidence events), experienced at least one acute event before study). Note that data regarding acute events before the study period was only collected for patients diagnosed before the start of the study period (i.e., 2008 or earlier).

Other primary outcomes will be analyzed descriptively, see **Section 3.6.1**.

3.6.3 Analysis of secondary outcomes

Treatment of cTTP

To address secondary objectives described in **Section 3.5.3**, treatment and related outcomes will be examined. Treatment will be reported separately for each event (acute event over entire study period or isolated TTP manifestation at index only). Prophylaxis treatment during the study period will also be described.

Healthcare Resource Utilization (HRU)

The following HRU-related outcomes will be described: Hospitalizations and specialists involved, which will be reported separately for each type of event (acute events during the study period or isolated TTP manifestation at index only). Specialist consultations for managing TTP related comorbidities will be reported for the entire study period.

3.7 Data Management

3.7.1 Missing data

Incomplete (i.e., missing) data will be reported, for all study variables. Characteristics at index will be reported for the full cohort. Study outcomes will be analyzed for those subjects with complete data. No imputation of missing responses will be conducted on the data.

3.7.2 Data cleaning

Prior to analysis, missing values will be recoded to be recognizable by the software used (Stata 17) using a list of system missing values provided by CISIV. Additionally, as part of our data quality checks, we will identify the use of other coding for missing values by sites. After confirming with the sites that the data is actually missing, those values will also be recoded to be recognized as missing in Stata. Sites will have the option to select "no data" in all sections. This means that the number of patients reporting data will vary dependent on the data availability.

3.7.3 Quality control

Quality control of the data will be undertaken by the statistician assigned to the project. The statistician will perform the analysis in accordance with this statistical analysis plan (SAP). Once each step in the SAP had been completed – the statistician will complete a code review and quality check. This quality control process will ensure:

- To check adherence to requirements and specifications as per the SAP
- To test and check the entire code including review of any errors in the log
- To evaluate interim data sample size (where applicable) and outcomes and confirm expected results

This stepwise approach will utilize an on-going quality control rather than a complete code review being conducted at the end of the project. This process will ensure that any changes or adjustments that needed to be made to the code can be completed at an early stage and maintain a controlled development process.

REFERENCES

1. Public Policy Committee ISoP. Guidelines for good pharmacoepidemiology practice (GPP). . Pharmacoepidemiology and drug safety 2016:2–10 25.
2. Kremer Hovinga JA, Coppo P, Lämmle B, Moake JL, Miyata T, Vanhoorelbeke K. Thrombotic thrombocytopenic purpura. Nat Rev Dis Prim. 2017:17020 3.
3. van Dorland HA, Taleghani MM, Sakai K, Friedman KD, George JN, Hrachovinova I, et al. The International Hereditary Thrombotic Thrombocytopenic Purpura Registry: Key findings at enrollment until 2017. Haematologica. 2019:2107–15 104.
4. George JN. The thrombotic thrombocytopenic purpura and hemolytic uremic syndromes: evaluation, management, and long-term outcomes experience of the Oklahoma TTP-HUS Registry, 1989-2007. Kidney Int Suppl 2009:S52-4.
5. Scully M, Blombery P. Management of thrombotic thrombocytopenic purpura: current perspectives. J Blood Med. 2014;15:5.
6. Krogh AS von, Waage A, Quist-Paulsen P. Congenital thrombotic thrombocytopenic purpura. 2016:1452–7 136m.
7. Scully M, Hunt BJ, Benjamin S, Liesner R, Rose P, Peyvandi F, et al. Guidelines on the diagnosis and management of thrombotic thrombocytopenic purpura and other thrombotic microangiopathies. Br J Haematol 2012:p. 323–35. 2012.
8. Coppo P, Wolf M, Veyradier A, Bussel A, Malot S, Millot GA, et al. Prognostic value of inhibitory anti-ADAMTS13 antibodies in adult-acquired thrombotic thrombocytopenic purpura. Br J Haematol. 2006:66-74 132.
9. Ferrari S, Scheiflinger F, Rieger M, Mudde G, Wolf M, Coppo P, et al. Prognostic value of anti-ADAMTS 13 antibody features (Ig isotype, titer, and inhibitory effect) in a cohort of 35 adult French patients undergoing a first episode of thrombotic microangiopathy with undetectable ADAMTS 13 activity. Blood. 2007:2815–22 109.

APPENDIX

Table 10: Precipitating factors

Precipitating factors
SLE; Sjogren's; Addison's disease; Antiphospholipid syndrome; Connective tissue disease; Seronegative arthritis; Diabetes; Other autoimmune disease; Infection; Pregnancy; Mitomycin C; Cyclosporine; Quinine; Clopidogrel; Ticlopidine; Hormonal contraceptives; Vaccinations; Excessive alcohol consumption; HIV; Pancreatitis; Surgery; Trauma; Inflammation; Other.

HIV: Human Immunodeficiency Virus; SLE: Systemic Lupus Erythematosus

Table 11: Treatments

Acute events and isolated TTP manifestations
PEX; FFP; SDP; CSP; Hemodialysis; Peritoneal dialysis; Hemofiltration; Hemodiafiltration; BPL 8Y; Koate; Alphanate; Other.
Prophylaxis
Koate; Alphanate; 8Y; PEX; FFP; SDP; CSP; Regular dialysis; Prednisolone; Cyclosporine A; Azathioprine; Mycophenolate Mofetil (MMF).

PEX: Plasma exchange; FFP: Fresh frozen plasma; SDP: Solvent/detergent-treated plasma; CSP: Cryosupernatant plasma.

Table 12: Treatment related complications (for PEX, FFP and CSP only)

Treatment related complications (for PEX, FFP and CSP only)
Fever, chills; Muscle cramps; Hypotension; Nausea/vomiting; Abdominal pain; Chest pain; Dyspnea; Convulsions; Respiratory arrest; Allergic reaction; Anaphylaxis; Headache; Itching; Hemolytic transfusion reactions; Sepsis; Blood-borne viral infections; Post-transfusion purpura; Transfusion related acute lung injury (TRALI); Fluid overload; Nausea/vomiting; Transfusion-related graft versus host disease; Not applicable; Other.

Table 13: Treatment frequency

Frequency of administrations
Once Per Day (QD); Twice Per Day (BID); Three Times a Day (TID); Four Times a Day (QID); As Needed (PRN); At Bedtime, Nightly (QHS); Five Times Daily (5XD); Six Times Daily (6XD); Every Other Day (QOD); Every Other Week (QOW); Once Weekly (QS); Twice Weekly (BIS); Thrice Weekly (TIS); One time only; Other.

Table 14: Specialties

Specialties
Cardiologist; Hepatologist; Nephrologist; Hematologist; Gastroenterologist; Gynecologist; Ophthalmologist; Physiotherapist; Nurse specialists; TTP specialist center; Neurologist; Psychologist; Psychiatrist; Social worker; Transfusionist; Other.

Table 15: Comorbidities

Comorbidities
Other circulatory disease; Epilepsy; Amyotrophic Lateral Sclerosis (ALS); Dementia; Alzheimer's disease; Anxiety; Attention deficit (hyperactivity) disorder / AD(H)D; Autism spectrum disorder; Obsessive Compulsive Disorder; Depression; Drug Dependence; Alcohol Dependence; Post-traumatic stress disorder; Other mental or behavioral disorder; Gingivitis; Obesity; Type I Diabetes; Type II Diabetes mellitus; Osteoarthritis; Osteoporosis; Other musculoskeletal disorder; HIV; Systemic Lupus Erythematosus ; Sjogren's disease; Addison's disease; Antiphospholipid antibody syndrome; Seronegative arthritis; Hepatitis A; Hepatitis B; Hepatitis C; Cirrhosis; Other; None.

HIV: Human Immunodeficiency Virus.

Table 16: Symptoms

**Category**	**Symptoms**
Systemic manifestations and prodromes	DIC; Weakness/fatigue; Pallor; Fever; Dyspnea; Headache; Upper respiratory tract infection; Other
Neurological manifestations	Headache; Dizziness; Confusion/agitation; Stroke; Coma; Seizures; Febrile Seizures; Visual Disturbances (Phosphenes); TIA; Dysphasia; Dysarthria; Paranesthesia; Paresis; Personality Change; Motor Deficit; Dysphonia; Other
Gastrointestinal / hepato-pancreato-biliary (GI/HPB)	Abdominal Pain; Nausea/vomiting; Diarrhea; HPB issues; Gastrointestinal Hemorrhage; Other
Cardiovascular manifestations	ECG abnormalities; AMI (acute myocardial infarction)/ Ischemic heart disease; AF (atrial fibrillation); Congestive heart failure; Chest pain; Palpitations; Arrhythmia; Hypertension; Vasculopathy; Thrombosis; Angina; Other
Renal manifestations	Serum creatinine >1.5 x baseline; Proteinuria / hematuria; Oliguria; Acute Kidney injury; Other
Ophthalmic involvement	Retinal detachment; Retinal hemorrhage; Arterial or venous occlusion; Other
Pulmonary involvement	Pleural effusion; Tachypnoea; Pulmonary Oedema; Embolism; Other
Bleeding	Epistaxis; Intracranial hemorrhage; GI bleeds; GU bleeds (hematuria); Meno-metrorrhagia; Skin hemorrhage; Mucosal hemorrhage; Other
Musculoskeletal involvement	Rhabdomyolysis; Myalgia; Other
Skin	Maculopapular rash; Ulcer; Other

DIC: disseminated intravascular coagulation; ECG: echocardiogram; GU: genitourinary; TIA: transient ischemic attack

Table 17: Pregnancy-related complications

Pregnancy-related complications
Gestational thrombocytopenia; Gestational diabetes; Hyperemesis gravidarum; HELLP; Pre-eclampsia; Renal impairment; Transient Ischemic Attack (TIA); Disseminated Intravascular Coagulation (DIC); Venous Thromboembolism (VTE); Iron Deficiency Anemia (IDA); Cardiac symptoms; Placental abruption; Hypertension; Birth defects; Other obstetric complications; Early miscarriage (<12 weeks); Late miscarriage (>12 weeks); Stillbirth; Intrauterine fetal death; Ectopic pregnancy; Death of patient; None

SUPPLEMENTARY MATERIALS

Methods

Index events

Index events are defined in **Table S1**. The first of any of these events to occur within the index identification period (January 1, 2009–December 31, 2017) was considered the index event.

Table S1. Index Events

Index Event	Definition
Diagnosis of cTTP	Patients who met any of the following criteria were considered to have a documented diagnosis of severe hereditary ADAMTS13 deficiency:<br>• Diagnosis of cTTP confirmed by molecular genetic testing or documented in the patient's medical history, and<br>• Severe ADAMTS13 deficiency (ADAMTS13 activity ≤10% of normal) in the absence of an ADAMTS13 inhibitor in plasma samples drawn at 2 different time points no more than 14 days apart<br>o Patients receiving prophylactic PBT may exceed ADAMTS13 activity as documented in the patient's medical history, and/or<br>• Confirmed cTTP diagnosis based on other criteria specified by the healthcare professional (locally used criteria)
Acute TTP event	Defined as a drop in platelet count ≥50% of baseline or a platelet count <100,000/µL and an elevation of LDH >2× the ULN
TTP manifestation	Defined as events meeting ≥1 of the following criteria:<br>• Thrombocytopenia: a drop in platelet count ≥25% of baseline at screening or a platelet count <150,000/µL<br>• Microangiopathic hemolytic anemia, including elevation of LDH >1.5× ULN<br>• Relevant signs and symptoms according to the opinion of the site investigator or treating physician including:<br>o Other neurologic symptoms (eg, confusion, memory issues, irritability, dysarthria, focal or general motor symptoms including seizures, headache)<br>o Lethargy<br>o Abdominal pain<br>o Increase in serum creatinine >1.5× baseline
TTP-related clinical event	Defined as any of the following:<br>• Cerebrovascular event<br>• Cardiovascular event<br>• Thrombosis<br>• Organ-related event<br>• Gastrointestinal event<br>• Bleeding<br>• Neurologic event<br>• Any other acute clinical event related to cTTP<br>Events were recorded based on the physician's assessment

Receipt of prophylactic treatment for prevention of TTP-related events or regular PBT prophylaxis	Prevention of TTP-related events defined as the administration of prophylactic treatment<br>Regular PBT prophylaxis defined as prophylaxis with either fresh frozen plasma, plasma treated with a solvent-detergent process, or plasma-derived factor VIII-VWF concentrates based on prescribed/scheduled frequency. Frequency should be at least once per month and up to 3 times per week

ADAMTS13, a disintegrin and metalloproteinase with thrombospondin motifs 13; cTTP, congenital thrombotic thrombocytopenic purpura; LDH, lactate dehydrogenase; PBT, plasma-based therapy; TTP, thrombotic thrombocytopenic purpura; ULN, upper limit of normal.

Study variables

Sociodemographic variables included age, sex, race-ethnicity, country of residence. General health factors included body mass index, smoking, alcohol dependence, and drug dependence. cTTP-related clinical characteristics included the index event and symptomatology, and diagnostic history included age at diagnosis, family history, and diagnostic basis.

Eligibility criteria

The inclusion and exclusion criteria for patients included in the post hoc analysis are listed below:

- Patients had to be 0–70 years old
- For female patients who were ≥14 years old, data from prophylaxis use during pregnancy were excluded
- Patients were excluded if they had any of the following medical conditions: drug dependence, dementia, Alzheimer's disease, alcohol dependence, HIV, cirrhosis, or acute coronary syndrome
- Patients had to have received regular prophylaxis with fresh frozen plasma, solvent/detergent-treated plasma, or factor VIII products based on the prescribed/scheduled frequency (at least once per month and up to 3 times per week)

Sample size

Due to the rarity of cTTP, convenience sampling from the target population (i.e., patients with a confirmed diagnosis of cTTP) was employed to reach an estimated number of 80 patients. This was considered to be a meaningful target sample size for the study.

In total, 42 sites in Austria (1), France (2), Germany (7), Italy (3), Spain (9), Japan (1), Norway (1), Switzerland (1), the UK (3), and the US (14) were invited to participate in the study. Target patient numbers per country are shown in **Table S2**.

Table S2. Patient Numbers per Country

**Country**	**Target No. of Patients**	**Final No. of Patients Included**
Japan	10	0
USA	14	1
UK	7	8
Italy	7	2
France	7	50
Germany	7	5
Spain	7	6
Switzerland	9	6
Austria	6	0
Norway	6	0
**Total**	**80**	**78**

Nine sites were unable to participate due to the global response to the COVID-19 pandemic. The site in Japan did not report any eligible patients, and none of the sites in Austria or Norway participated in the study. The final study population included 78 patients from 9 sites across 7 countries: France (1), the UK (2), Spain (2), Switzerland (1), Germany (1), Italy (1), and the United States (1).

Table S3. Demographics and Clinical Characteristics for Patients from France

**Characteristics**	**All Patients from France (N = 50)**
Sex, n (%)	
Female	41 (82.0)
Male	9 (18.0)
Age range at index event, n (%)	
<18 y	12 (24.0)
18-29 y	21 (42.0)
30-44 y	15 (30.0)
45-64 y	2 (4.0)
≥65 y	0
Age range at diagnosis, n (%)	
<18 y	16 (32.0)
18-29 y	19 (38.0)
30-44 y	13 (26.0)
45-64 y	2 (4.0)
≥65 y	0

Table S4. Index Event by Country

Country	Total No. of Patients	Diagnosis, n (%)	Diagnosis/ Acute TTP Event, n (%)	Acute TTP Event, n (%)	Prophylaxis, n (%)
**Total**	**78**	**35 (44.9)**	**28 (35.9)**	**18 (23.1)**	**16 (20.5)**
France	50	19 (38.0)	14 (28.0)	10 (20.0)	13 (26.0)
Germany	5	2 (40.0)	2 (40.0)	3 (60.0)	0
Italy	2	1 (50.0)	0	1 (50.0)	0
Spain	6	4 (66.7)	4 (66.7)	1 (16.7)	1 (16.7)
Switzerland	6	5 (83.3)	4 (66.7)	1 (16.7)	0
UK	8	4 (50.0)	4 (50.0)	2 (25.0)	1 (12.5)
US	1	0	0	0	1 (100)

Diagnosis corresponding with an acute TTP event is listed as diagnosis/acute TTP event

Denominator is the total number of patients for each country.

Table S5. Receipt of Prophylaxis by Country.

Country	Total No. of Patients	Patients with Prophylaxis[1], n (%)	No Prophylaxis[2], n (%)
**Total**	**78**	**47 (60.3)**	**31 (39.7)**
France	50	34 (68.0)	16 (32.0)
Germany	5	0	5 (100)
Italy	2	1 (50.0)	1 (50.0)
Spain	6	2 (33.3)	4 (66.7)
Switzerland	6	2 (33.3)	4 (66.7)
UK	8	7 (87.5)	1 (12.5)
US	1	1 (100)	0

[1] Patients with at least one prophylaxis period. Denominator is the total number of patients for each country.

[2] Patients who have no record of prophylaxis. Denominator is the total number of patients for each country.

Supplementary Figure S1. Study Design Schematic

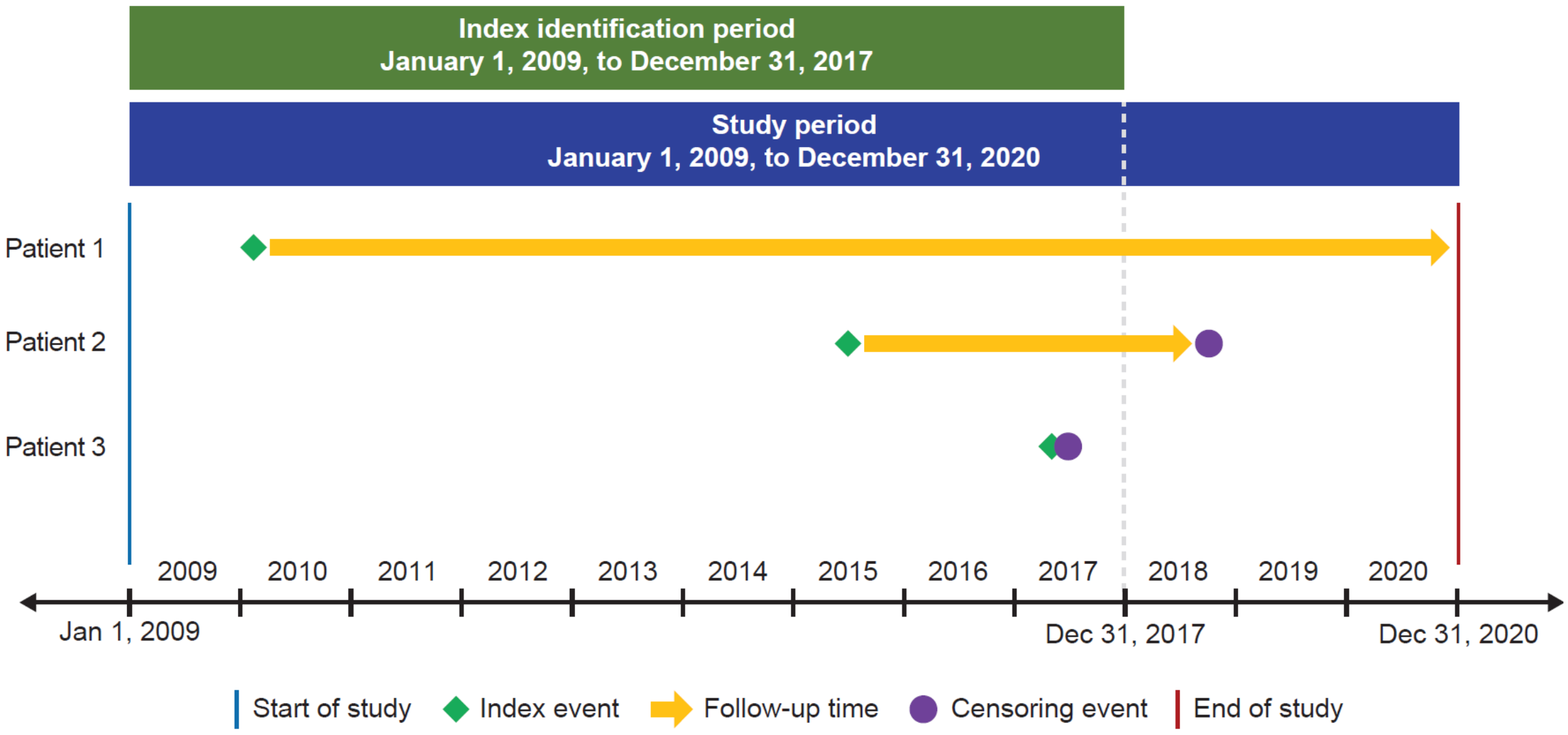

Patients 1, 2, and 3 are hypothetical patients. Possible censoring events: loss to follow-up, enrollment in a clinical trial, or death.

Supplementary Figure S2. Flowchart to Identify the Subset of Patients With cTTP Receiving Regular Prophylaxis

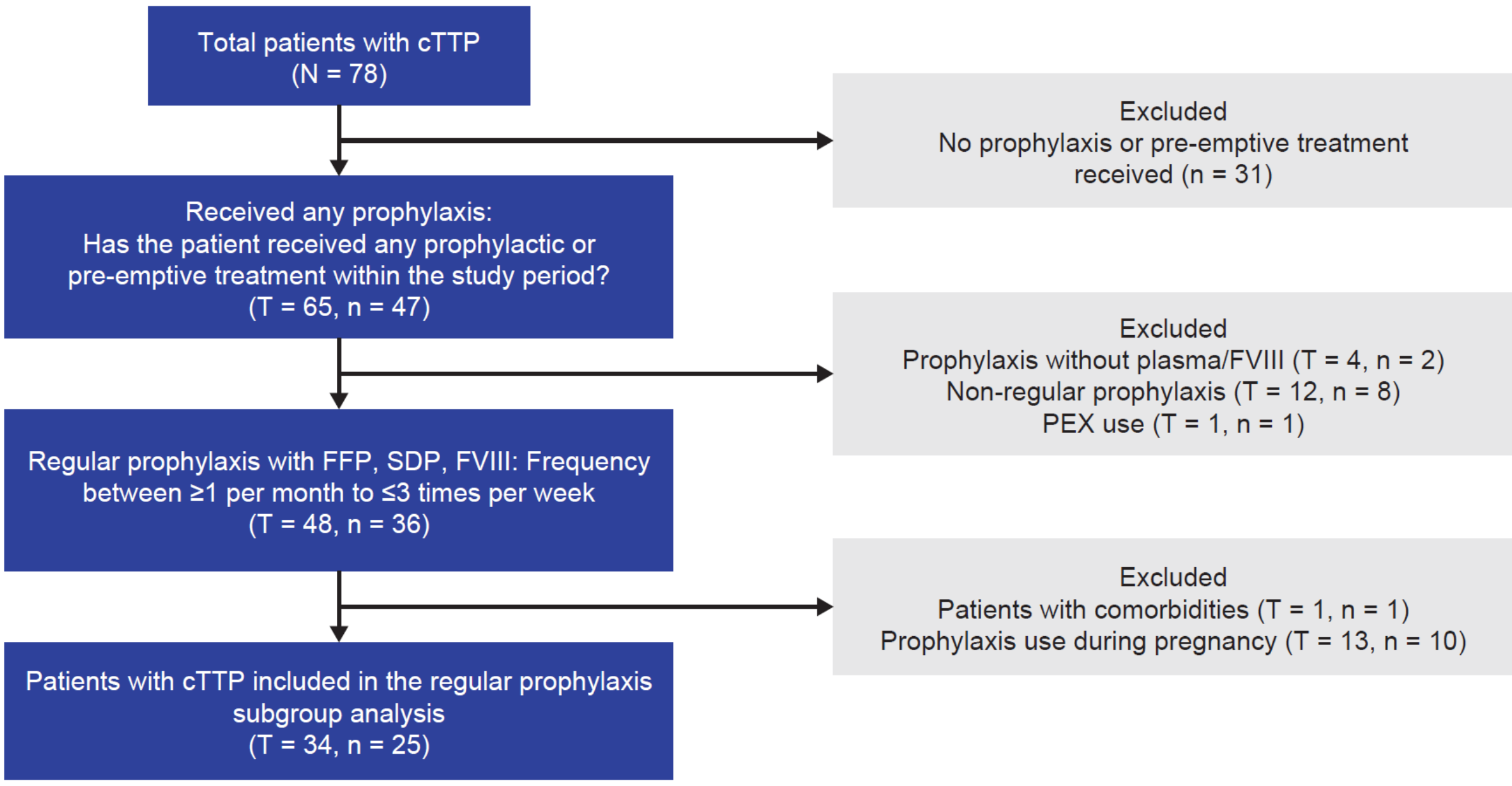

Abbreviations: cTTP, congenital thrombotic thrombocytopenic purpura; FFP, fresh frozen plasma; FVIII, factor VIII; PEX, plasma exchange; SDP, solvent/detergent-treated plasma; T, number of treatments.

STROBE Statement—Checklist of items that should be included in reports of ***cohort studies***

	Item No	Recommendation	Page No
**Title and abstract**	1	(*a*) Indicate the study's design with a commonly used term in the title or the abstract	1
		(*b*) Provide in the abstract an informative and balanced summary of what was done and what was found	1-2
**Introduction**			
Background/rationale	2	Explain the scientific background and rationale for the investigation being reported	3-4
Objectives	3	State specific objectives, including any prespecified hypotheses	4
**Methods**			
Study design	4	Present key elements of study design early in the paper	4-5
Setting	5	Describe the setting, locations, and relevant dates, including periods of recruitment, exposure, follow-up, and data collection	4-5
Participants	6	(*a*) Give the eligibility criteria, and the sources and methods of selection of participants. Describe methods of follow-up	6
		(*b*) For matched studies, give matching criteria and number of exposed and unexposed	N/A
Variables	7	Clearly define all outcomes, exposures, predictors, potential confounders, and effect modifiers. Give diagnostic criteria, if applicable	6-7
Data sources/ measurement	8*	For each variable of interest, give sources of data and details of methods of assessment (measurement). Describe comparability of assessment methods if there is more than one group	6-9
Bias	9	Describe any efforts to address potential sources of bias	4-9
Study size	10	Explain how the study size was arrived at	8
Quantitative variables	11	Explain how quantitative variables were handled in the analyses. If applicable, describe which groupings were chosen and why	7-8
Statistical methods	12	(*a*) Describe all statistical methods, including those used to control for confounding	7-8
		(*b*) Describe any methods used to examine subgroups and interactions	
		(*c*) Explain how missing data were addressed	
		(*d*) If applicable, explain how loss to follow-up was addressed	
		(*e*) Describe any sensitivity analyses	
**Results**			
Participants	13*	(a) Report numbers of individuals at each stage of study—eg numbers potentially eligible, examined for eligibility, confirmed eligible, included in the study, completing follow-up, and analysed	9-10
		(b) Give reasons for non-participation at each stage	
		(c) Consider use of a flow diagram	
Descriptive data	14*	(a) Give characteristics of study participants (eg demographic, clinical, social) and information on exposures and potential confounders	9-10
		(b) Indicate number of participants with missing data for each variable of interest	9-14
		(c) Summarise follow-up time (eg, average and total amount)	9
Outcome data	15*	Report numbers of outcome events or summary measures over time	9-14

Main results	16	(*a*) Give unadjusted estimates and, if applicable, confounder-adjusted estimates and their precision (eg, 95% confidence interval). Make clear which confounders were adjusted for and why they were included<br>(*b*) Report category boundaries when continuous variables were categorized<br>(*c*) If relevant, consider translating estimates of relative risk into absolute risk for a meaningful time period	9-14
Other analyses	17	Report other analyses done—eg analyses of subgroups and interactions, and sensitivity analyses	13-14
**Discussion**			
Key results	18	Summarise key results with reference to study objectives	14-20
Limitations	19	Discuss limitations of the study, taking into account sources of potential bias or imprecision. Discuss both direction and magnitude of any potential bias	18-19
Interpretation	20	Give a cautious overall interpretation of results considering objectives, limitations, multiplicity of analyses, results from similar studies, and other relevant evidence	14-20
Generalisability	21	Discuss the generalisability (external validity) of the study results	19
**Other information**			
Funding	22	Give the source of funding and the role of the funders for the present study and, if applicable, for the original study on which the present article is based	21

*Give information separately for exposed and unexposed groups.

Note: An Explanation and Elaboration article discusses each checklist item and gives methodological background and published examples of transparent reporting. The STROBE checklist is best used in conjunction with this article (freely available on the Web sites of PLoS Medicine at http://www.plosmedicine.org/, Annals of Internal Medicine at http://www.annals.org/, and Epidemiology at http://www.epidem.com/). Information on the STROBE Initiative is available at http://www.strobe-statement.org.
